# Computer-Aided Diagnosis System for Sow Pregnancy Using Low-Frequency Portable Ultrasound Videos with Preprocessing and Augmentation Techniques

**DOI:** 10.3390/ani16213470

**Published:** 2026-11-03

**Authors:** Yo Han Choi, Jae-beom Park, Tae-kyeong Kim, Hyeong-sik Won, Hyun Ju Park, Hong-jun Kim, Jo Eun Kim, Yong Dae Jeong, Jin-soo Kim, Hyun-sik Ham, Hyun-chong Cho

**Affiliations:** 1 Swine Science Division, National Institute of Animal Science, Rural Development Administration, Cheonan 31000, Republic of Korea; cyh6150@korea.kr (Y.H.C.); 77qk77@naver.com (H.J.P.); hjkim93@korea.kr (H.-j.K.); kjektw@korea.kr (J.E.K.); yongdaejeong@korea.kr (Y.D.J.); 2 Interdisciplinary Graduate Program for BIT Medical Convergence, Kangwon National University, Chuncheon 24341, Republic of Korea; qkrwoqja100@kangwon.ac.kr; 3 Department of Data Science, Kangwon National University, Chuncheon 24341, Republic of Korea; rlaxorud0219@kangwon.ac.kr (T.-k.K.); sikwkd1118@kangwon.ac.kr (H.-s.W.); 4 College of Animal Life Sciences, Kangwon National University, Chuncheon 24341, Republic of Korea; kjs896@kangwon.ac.kr; 5 Department of Electronics Engineering, Kangwon National University, Chuncheon 24341, Republic of Korea

**Keywords:** computer-aided diagnosis, data augmentation, deep learning, image preprocessing, low-frequency portable ultrasound video, sow pregnancy diagnosis

## Abstract

Accurate pregnancy diagnosis is important for reproductive management in pig farming. This study evaluated a computer-aided diagnosis workflow using portable low-frequency ultrasound videos from 1895 sows at two sites. Xception frame predictions were aggregated into video-level decisions. Original, Preprocessing (wavelet denoising and contrast-limited adaptive histogram equalization), Mixup, and their combination (Combined) were compared across ten repeated sow-level splits. Combined achieved mean accuracies of 84.5% and 85.3% in Datasets A and B, respectively, with pregnant-sow videos restricted to days 18–25 post-insemination. Compared with Original, mean specificity increased by 1.3 and 2.4 percentage points and Youden’s index by 1.7 and 2.3 points, while mean sensitivity was numerically similar. Paired 95% confidence intervals for specificity and Youden’s index differences excluded zero only in Dataset B, without adjustment for multiple comparisons. Paired comparisons of the area under the curve (AUC) did not establish improved discrimination. Mixup yielded the highest mean specificity and AUC, whereas Combined yielded higher mean sensitivity than Mixup. This trade-off varied across partition subsets. Cross-site and leave-one-session-out evaluations showed performance variability across acquisition settings, with no consistently best condition. These comparisons identify configuration-dependent trade-offs requiring further evaluation for diagnostic support.

## Simple Summary

Early and accurate pregnancy diagnosis is important for breeding management and farm productivity. Many farms use portable, low-cost ultrasound devices that produce images of limited quality. Using videos from 1895 sows at two sites, we compared a computer-based diagnostic workflow with no additional image processing or training-data augmentation, image processing alone, training-data augmentation alone, and their combination. The workflow combines predictions from individual images into a video-level decision. Across ten repeated evaluations, the combined approach yielded numerically higher average percentages of correctly classified videos from nonpregnant sows than the unmodified approach: 74.8% versus 73.5% at one site and 85.4% versus 83.0% at the other. Statistical evidence for this difference was found only at the second site. The ability to identify pregnant sows was numerically similar between these approaches. Considering pregnant and nonpregnant videos together, the combined approach achieved an overall accuracy of approximately 85% at both sites. However, performance varied across sites and recording sessions, and no approach performed best in every setting. These comparisons identify trade-offs among the approaches and support further evaluation to determine whether the tool can assist specialists with pregnancy diagnosis on farms.

## 1. Introduction

**Figure 1:**
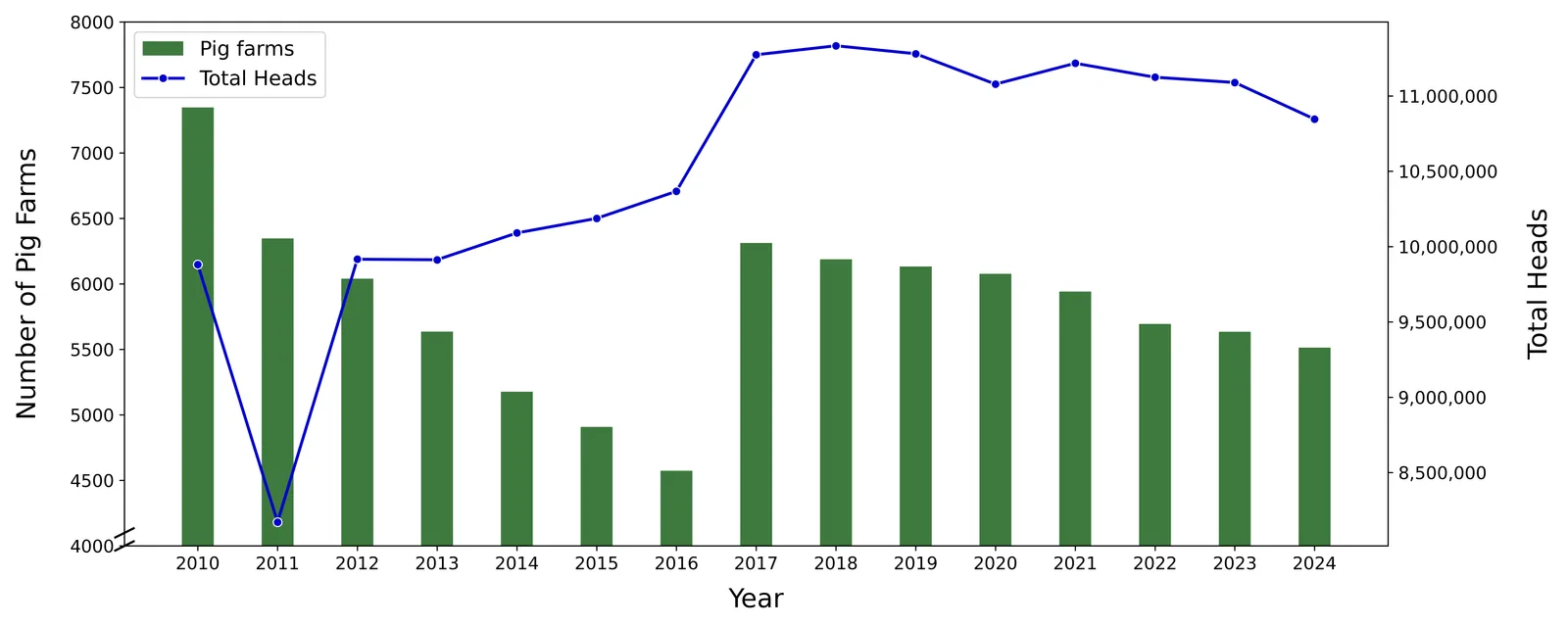
Number of pig farms and pig population in the Republic of Korea from 2010 to 2024 (data from [7]).

Effective management of pig reproduction is intrinsically linked to the overall productivity of pig farms [1,2,3]. Early identification of pregnant sows facilitates timely and appropriate nutritional management. Consequently, various approaches to early pregnancy detection have been explored. Traditional methods for pregnancy diagnosis include monitoring behavioral and physical changes, measuring hormone levels, and conducting ultrasound examinations [4,5,6]. The workload associated with pregnancy diagnosis may increase as the number of pigs managed per farm grows. Between 2010 and 2024, the pig population in the Republic of Korea increased while the number of farms decreased; consequently, the average number of pigs per farm rose [7]. Figure 1 shows these national trends. These trends underscore the need for rapid, objective diagnostic support compatible with low-cost devices. In this context, low-frequency portable ultrasound devices can offer a practical option because of their relatively low cost and portability. However, the limited quality of ultrasound images obtained using such devices can make early pregnancy diagnosis more difficult.

Recent advances in deep learning have enabled automated analysis of ultrasound images and videos. In fetal ultrasound, frame-level convolutional networks have been used to detect and localize standard scan planes in freehand video in real time [8]. In echocardiography, Howard et al. compared frame-based convolutional neural network (CNN) classification with architectures that incorporate temporal information and reported lower view-classification error with the best-performing two-stream model [9]. These studies provide methodological context for the present study, although their clinical tasks differ from sow pregnancy diagnosis.

Speckle noise can reduce image contrast and complicate ultrasound image interpretation. Thus, speckle reduction in ultrasound images has been widely investigated using Bayesian multiscale estimation [10], nonlocal means filtering [11], and deep residual learning [12]. Although these studies reported improvements in image quality metrics, improved image quality does not inherently translate into better classification performance. This motivates an empirical evaluation of whether denoising substantially benefits pregnancy diagnosis models.

Numerous institutions and researchers have been actively exploring deep- and machine-learning technologies to support automation in swine management. Chang et al. [13] employed the You Only Look Once version 4 (YOLOv4) model to automatically calculate sow vulva size and monitor changes for estrus detection. Additionally, Yu et al. [14] and Li et al. [15] conducted studies measuring backfat thickness, which is highly correlated with lifespan and reproductive performance in sows.

More specifically, studies have investigated approaches to sow pregnancy diagnosis. Gokuldas et al. [16] evaluated the diagnostic accuracy and effectiveness of real-time ultrasound imaging for pregnancy diagnosis. Martinez et al. [17] investigated the use of ultrasound technology for pregnancy diagnosis and detection of reproductive failure. Zhou et al. [18] identified circulating exosomal microRNAs as potential biomarkers for early pregnancy diagnosis in sows. Kousenidis et al. [19] trained a deep neural network to predict litter size from numerical features derived from real-time ultrasound information. Schwier et al. [20] developed a region-based method for detecting early-stage sow pregnancy from ultrasound images during days 22–25 post-insemination. Relative to Schwier et al. [20], the present study extends the evaluation window to include days 18–21 post-insemination. More recently, Kim et al. [21] compared ViT-H, ConvNeXt-Xlarge, and Xception with AutoAugment for image-based sow pregnancy classification across several gestational periods, including days 18–21. In contrast, the present study evaluates video-level decisions by aggregating frame predictions from videos of 1895 sows at two sites. Specifically, we examine the effects of wavelet denoising, contrast-limited adaptive histogram equalization (CLAHE), Mixup and their integrated combinations on classification performance.

This study aimed to compare preprocessing and augmentation strategies within a computer-aided diagnosis (CADx) workflow for early sow pregnancy diagnosis from B-mode 3.5 MHz low-frequency ultrasound videos. The CADx system employed Xception to provide frame-level predictions, which were aggregated into video-level decisions.

Within this workflow, wavelet denoising and CLAHE were evaluated to address noise and limited contrast, and Mixup was evaluated as a training-data augmentation strategy. Original, Preprocessing (Wavelet + CLAHE), Mixup and Combined (Wavelet + CLAHE + Mixup) were compared to assess whether these preprocessing and augmentation strategies improved classification and how they affected sensitivity and specificity. The main analysis used ten repeated sow-level train-validation-test splits; supplementary model and processing comparisons used three shared splits with matched Xception results. Cross-site testing and session-held-out evaluation in both datasets assessed performance beyond the repeated sow-level random splits. These analyses were intended to characterize the system’s performance and limitations.

## 2. Materials and Methods

To develop and validate the proposed CADx system, ultrasound videos of pregnant and nonpregnant sows were acquired by experienced technicians at two locations between February 2023 and May 2024. Videos from pregnant sows were included only when acquired 18–25 days post-insemination. The ultrasound device used for video acquisition in this study is described in Table 1.

The devices used in this study are portable B-mode scanners equipped with a 3.5 MHz abdominal sector probe and record videos at 1120 × 624 pixels. A 3.5 MHz sector probe has previously been evaluated for transabdominal pregnancy diagnosis in sows [22].

### 2.1. Dataset

The first dataset, referred to as Dataset A, comprised low-frequency portable ultrasound videos collected from pig farms operated by the College of Animal Life Sciences at Kangwon National University, Republic of Korea. These data were collected over six sessions. The second dataset, referred to as Dataset B, included low-frequency portable ultrasound videos obtained from pig farms managed by the National Institute of Animal Science (NIAS), Republic of Korea. These data were collected over six sessions.

The analytical datasets comprised 1484 ultrasound videos from 492 sows in Dataset A and 3938 videos from 1403 sows in Dataset B. Each sow contributed 1–4 videos in Dataset A and 1–6 videos in Dataset B, with mean ± standard deviation (SD) values of 3.0 ± 0.3 and 2.8 ± 0.5 videos per sow, respectively. In Dataset A, 458 of the 492 sows contributed three videos each, accounting for the small SD despite the range of 1–4 videos. Videos were approximately 10 s long. Study videos from each sow were acquired at a single time point; no sow contributed study videos acquired at different time points.

Dataset A shares 472 sows and 1428 source ultrasound videos with Kim et al. [21]. The shared acquisitions comprise sessions 1–4 and the nonpregnant acquisitions from session 5. Previously selected frames from these shared acquisitions were reused for model development in the present study. However, video-level testing used all available frames decoded from the held-out source videos rather than the previously selected frame sets. The shared acquisitions therefore do not constitute independent data relative to the previous study.

These datasets were used to train and evaluate the CADx system for sow pregnancy diagnosis. Pregnancy labels were provided by the participating farms following confirmation by an experienced swine reproduction specialist using follow-up ultrasound examinations. Pregnancy-status confirmation was based on serial ultrasound examinations performed as part of routine reproductive management at the participating farms, without a single fixed confirmation day. The records available to the research team did not document the interval from insemination to confirmation or establish the timing of the confirmatory examinations relative to study-video acquisition for each sow. Ultrasonographic pregnancy diagnosis in swine includes assessment of embryonic vesicles and developing conceptuses [23]. In the present study, a sow was classified as pregnant when an embryonic vesicle (gestational sac) was visualized during these examinations. Conversely, a sow was classified as nonpregnant when serial follow-up ultrasound examinations revealed no evidence of pregnancy after artificial insemination.

The personnel who acquired the study videos and the specialist who performed the confirmatory examinations were different. The same swine reproduction specialist conducted the serial follow-up examinations. All study videos were recorded with the device described in Table 1, whereas the follow-up examinations used the ultrasound devices in routine use on each farm, which may have differed from the study-video acquisition device. The video labels used in this study are the results of the follow-up examinations. Pregnancy-status labels were available for all included sows. Early embryonic loss was not assessed in this study, and its occurrence could not be determined from the study videos and the information available to the research team.

Acquisition-day information for nonpregnant videos was retrospectively reviewed using original video files and available acquisition and insemination records. In Dataset A, exact file-content matching to the original videos identified filename-recorded acquisition days for 27 of 152 nonpregnant records, comprising 81 of 459 videos: 12 records with 36 videos on days 15–17 and 15 records with 45 videos on days 18–25. In Dataset B, post-insemination intervals were reconstructed for 409 of 521 nonpregnant records, comprising 1129 of 1466 videos, by linking acquisition and insemination records using session and animal identifiers. These reconstructed intervals ranged from 11 to 29 days. Records without usable acquisition-day information were classified as Unknown: 125 records comprising 378 videos in Dataset A and 112 records comprising 337 videos in Dataset B. Sample counts by acquisition period are provided in Table S1.

For training and validation, every fifth frame was extracted with a maximum of 20 frames per video. Subsampling was used to reduce redundancy between adjacent frames, cap the contribution of each video at 20 training frames, and limit training memory and computation. Video-level testing did not use this subsampling (Section 2.5). The videos contained at most 100 frames (10 s at 10 frames/s), so the sampled training frames spanned the entire length of each video. After frame extraction, non-ultrasound regions, including text overlays, were removed using a single manually delineated binary mask. The mask was resized to each frame’s dimensions and applied using a bitwise AND operation. The same mask was also applied to frames decoded from the test videos. The details of these datasets are presented in Table 2 and Table 3.

Pregnancy-status labels were supplied by the data providers before frame extraction. After extracting every fifth frame, frames from pregnant-sow videos in Dataset A sessions 1–5 were reviewed to retain frames in which a gestational sac could be identified. The personnel performing this selection were aware of the pregnancy labels and were different from those who assigned the original video-level pregnancy-status labels. This selection was applied only to training frames from pregnant-sow videos; nonpregnant frames were not subjected to this filtering. It did not exclude entire videos and was not applied to Dataset A session 6, Dataset B or video-level testing. Dataset A sessions 1–5 were received together and processed during the initial data-preparation stage. The remaining data were received considerably later and did not undergo the same filtering because of time constraints. For the shared pregnant-sow acquisitions, this selection had been performed during the earlier data-preparation stage, and the resulting frames were reused in the present study.

### 2.2. Deep-Learning-Based Classification Architecture

**Figure 2:**
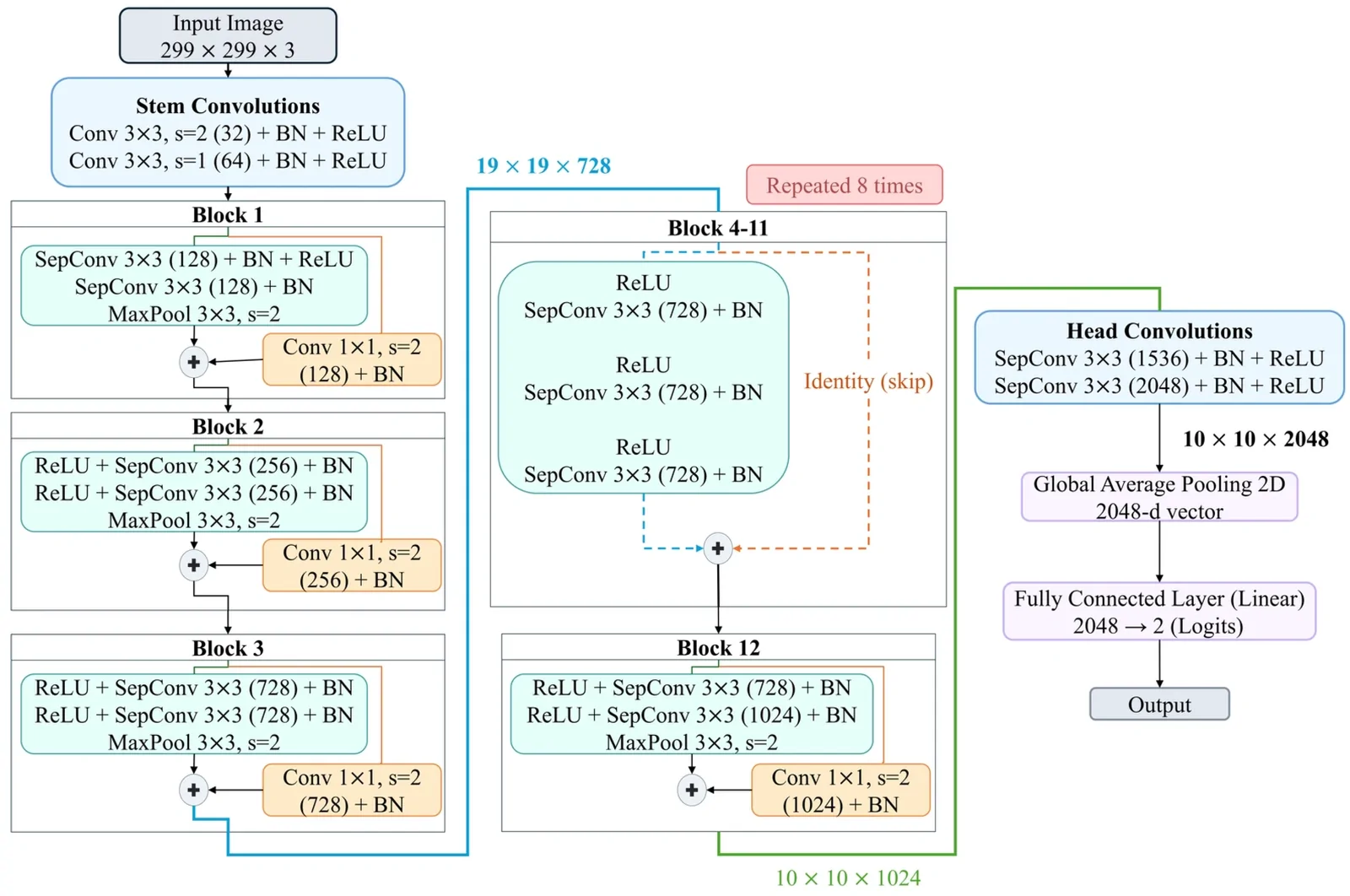
Xception-based classification architecture implemented in this study. Orange boxes and lines indicate 1 × 1 convolutional shortcuts with stride 2, orange dashed lines indicate identity shortcuts, and blue and green lines indicate connections between stages, labeled with the corresponding feature–map dimensions.

The Inception architecture uses 1 × 1 convolutions for channel reduction and max pooling for spatial downsampling [24]. Whereas the Inception architecture jointly processes cross-channel and spatial correlations, Xception separates the processing of these correlations through depthwise separable convolutions [25]. The Xception architecture is illustrated in Figure 2. A depthwise separable convolution consists of a depthwise convolution followed by a pointwise convolution. This separation can facilitate feature extraction while enabling more efficient use of model parameters. The use of Xception was also motivated by its performance in echocardiographic view classification, where it achieved the lowest error among the classical 2D CNNs evaluated in that study [9]. Ultrasound frames were resized from the 1120 × 624 processing dimensions to 299 × 299 pixels without cropping or padding, changing the aspect ratio. The ultrasound videos were decoded into three-channel images using OpenCV, without explicit replication of a single grayscale channel. Xception was initialized with ImageNet-pretrained weights and fully fine-tuned for binary classification using cross-entropy loss.

### 2.3. Image Preprocessing

Low-cost, low-frequency portable ultrasound equipment can produce images with noise and limited contrast, which may make classification more difficult. To mitigate noise, a wavelet-denoising algorithm was applied. A two-dimensional discrete wavelet transform was applied to the input image *f*(*x*,*y*) using a two-level Haar decomposition to obtain approximation and detail coefficients. The detail coefficients were processed using soft thresholding [26] with threshold *T* = *σ*/2, where *σ* denotes the standard deviation of the corresponding detail coefficients. This threshold was calculated directly from the detail coefficients rather than selected using universal thresholding or SURE-based optimization [27]. The denoised image was reconstructed using the inverse transform. Because the detail coefficients contain both image structure and noise, their standard deviation reflects both contributions rather than providing a noise-only estimate. Consequently, the threshold *T* = *σ*/2 depends on image content and should be regarded as a heuristic choice.

The Haar mother wavelet is defined in Equation (1):

(1) $$\psi \left(t\right)=\left\{\begin{array}{c} 1\;0\leq t<\frac{1}{2}\\ -1\;\frac{1}{2}\leq t<1\\ 0\;\mathrm{otherwise} \end{array}\right.$$

**Figure 3:**
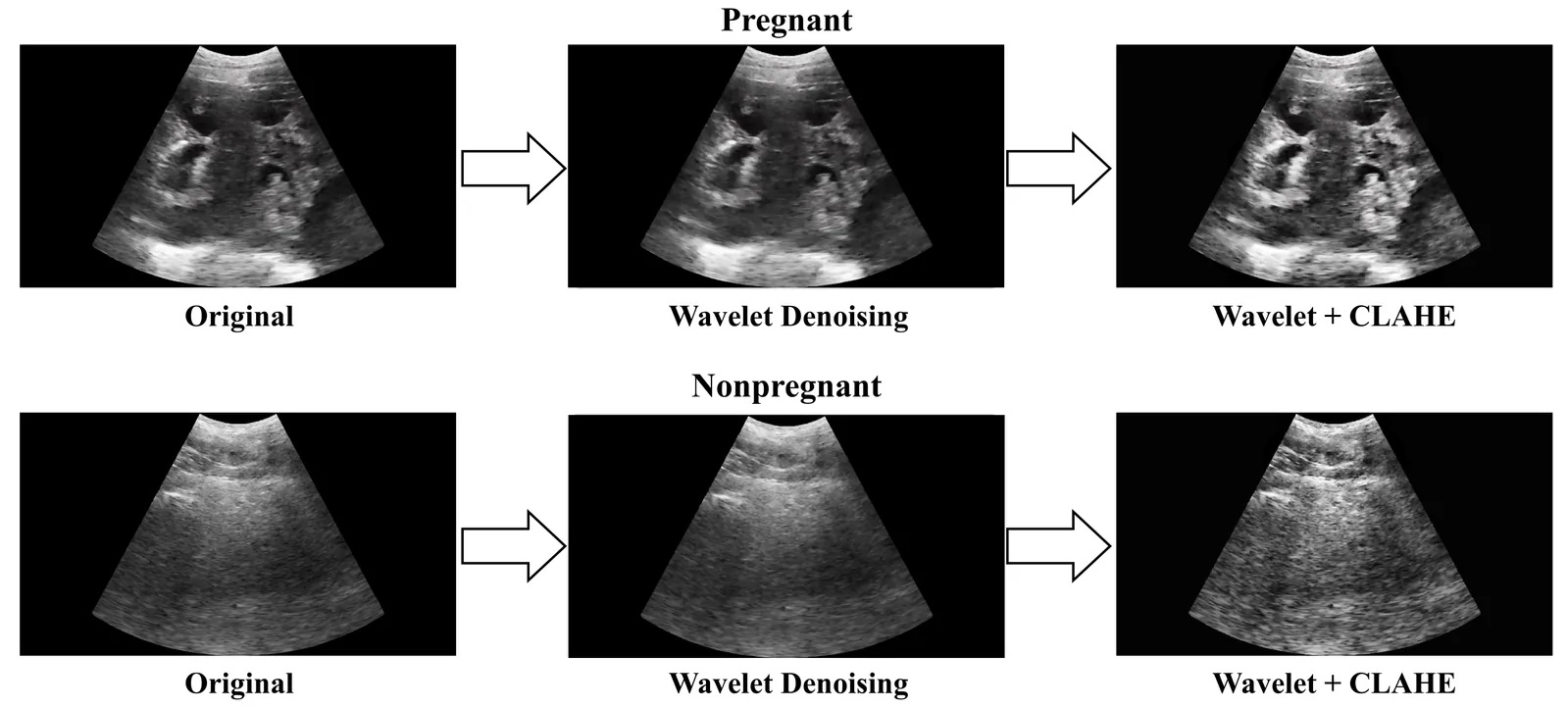
Examples of ultrasound images of pregnant and nonpregnant sows after the application of wavelet denoising and CLAHE.

Conventional histogram equalization adjusts the intensity distribution globally, which can lead to excessive contrast in some regions. Therefore, CLAHE was employed to limit excessive contrast enhancement during histogram equalization [28,29]. Specifically, it operates by dividing an image into small tiles and individually applying histogram equalization to each tile, resulting in more localized contrast enhancement. Thereafter, bilinear interpolation was employed between adjacent tiles to reduce abrupt changes in intensity. CLAHE was applied to the L channel in the LAB color space. In this study, the tile grid size was set to 8 × 8, which is the default of the OpenCV implementation, and the clip limit to 2, a commonly used value. Previous work has investigated the effects of CLAHE parameter choices on ultrasound-video enhancement [30]. Figure 3 shows examples of ultrasound images from pregnant and nonpregnant sows processed using wavelet denoising and CLAHE.

### 2.4. Mixup Algorithm

To improve performance on unseen data, data augmentation was employed. One such technique is Mixup, which linearly combines two images and their labels [31]. This approach was used to increase training data diversity. The generation of synthetic samples and labels using Mixup is expressed as follows:

(2) $$\tilde{x}=\lambda x_{i}+\left(1-\lambda \right)x_{j},$$

(3) $$\tilde{y}=\lambda y_{i}+\left(1-\lambda \right)y_{j},$$

where *x_i_* and *x_j_* denote two randomly selected training images, *y_i_* and *y_j_* denote their corresponding one-hot labels, $\tilde{x}$ and $\tilde{y}$ denote the mixed image and label, and *λ* ∈ [0,1] follows a Beta(*α*,*α*) distribution. In this study, *α* was set to 1, and the loss was computed according to the mixing coefficient *λ*. The corresponding loss function is expressed as follows:

(4) $$\tilde{L}=\lambda \cdot L\left(\hat{y},y_{i}\right)+\left(1-\lambda \right)\cdot L\left(\hat{y},y_{j}\right),$$

where $\hat{y}$ denotes the model prediction for the mixed image $\tilde{x}$. Mixup was applied only to the training frames after the sow-level data split; the validation and test data were not augmented. For the four main experimental conditions, Mixup was the only image augmentation method applied.

**Figure 4:**
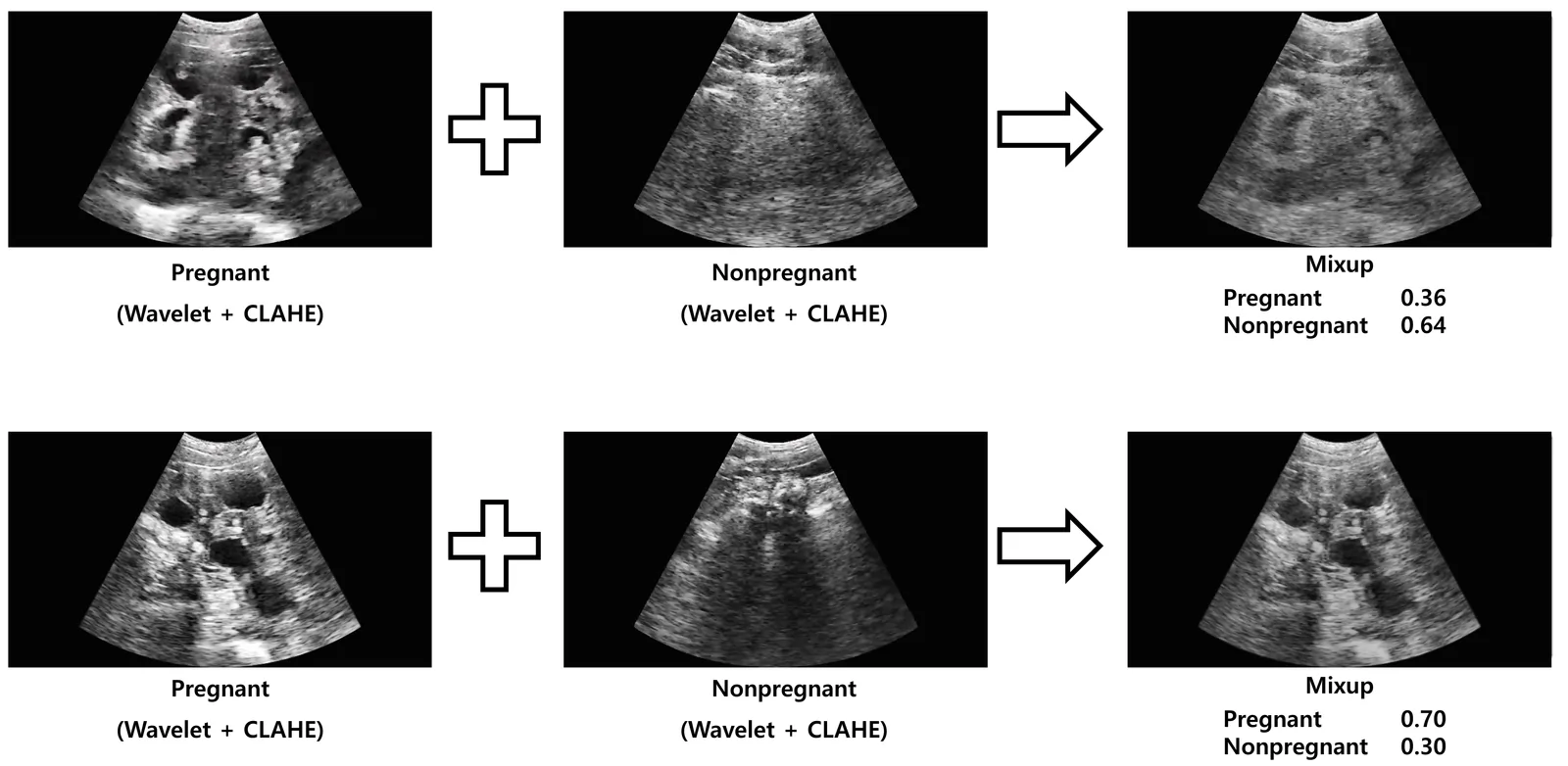
Examples of ultrasound images synthesized using the Mixup algorithm after the application of wavelet denoising and CLAHE.

Figure 4 shows examples of images from the Combined condition (Section 2.7), in which Mixup was applied after wavelet denoising and CLAHE.

### 2.5. Method for Video-Based Ultrasound Classification

The proposed method determines sow pregnancy status from low-frequency portable ultrasound videos. However, ultrasound images may inadvertently capture non-diagnostic content, such as the bladder and other tissues in addition to pregnancy-related structures, even when the sow is pregnant. To address this issue, an image-based video classification method was used to determine pregnancy status based on the proportion of frames classified as pregnant.

For video-level evaluation of held-out test videos, all available frames from each ultrasound video were used, and non-ultrasound regions were masked. For the Preprocessing and Combined conditions defined in Section 2.7, these frames were preprocessed using wavelet denoising and CLAHE to address noise and limited contrast. Each frame was then individually classified using the Xception model to predict pregnancy status. The proportion of frames predicted as pregnant was calculated. If the proportion was ≥0.5, the video was classified as pregnant; otherwise, it was classified as nonpregnant.

**Figure 5:**
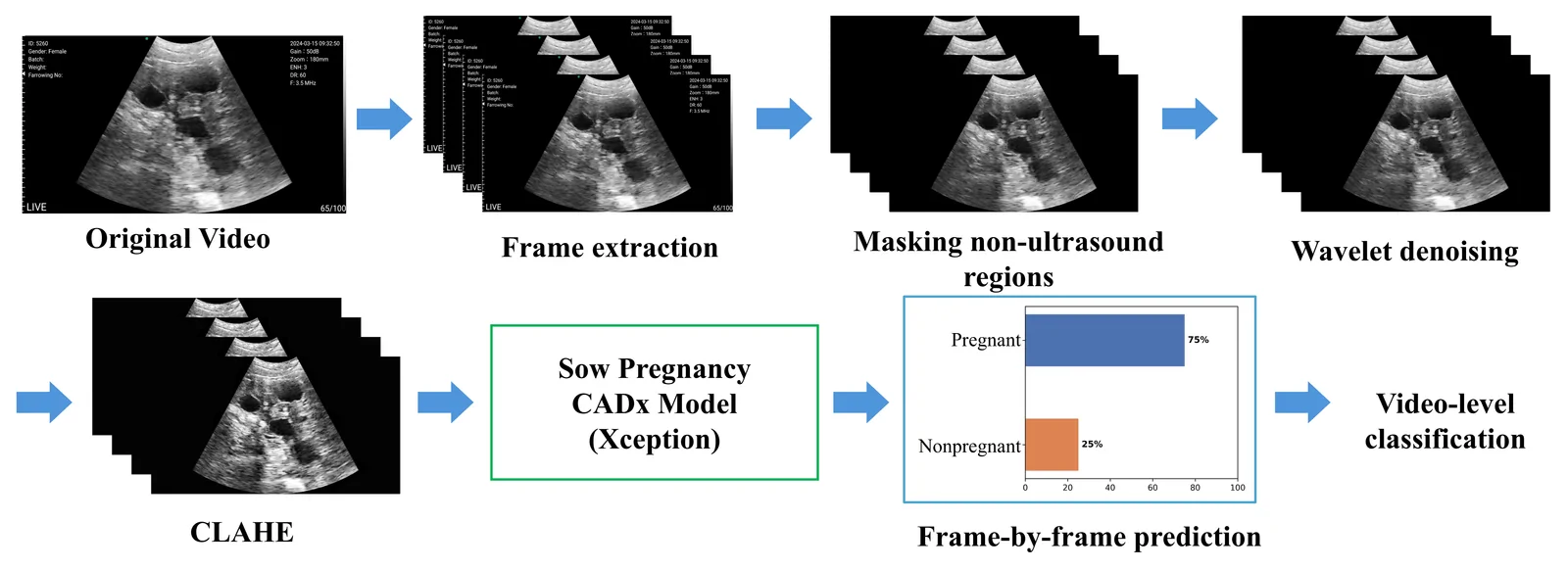
Frame-level prediction and aggregation workflow for video-level sow pregnancy classification. Wavelet denoising and CLAHE were applied only under Preprocessing and Combined. Mixup was used during training only and is not part of the inference workflow. A video was classified as pregnant when the proportion of frames predicted as pregnant was at least 0.5.

This method was intended to limit the influence of individual irrelevant frames on video-level classification. The prediction process of the method is illustrated in Figure 5.

The video-level threshold was prespecified at 0.5 and kept fixed across all datasets, experimental conditions and repetitions. Majority voting was adopted because it provides a straightforward decision rule without requiring additional tuning. Because the rule requires at least half of the frames to be classified as pregnant, a video containing only a small proportion of frames predicted as pregnant will be classified as nonpregnant. Alternative aggregation rules, defined below, are compared in Section 3.

For top-percentage pooling, pregnancy probabilities were obtained for all frames in each video and sorted in ascending order. Frames with the highest probabilities were selected, and their probabilities were averaged to obtain a video-level score. A video was classified as pregnant when this score was at least 0.5. The selected proportions were 10%, 20% and 30%, with the number of selected frames rounded to the nearest integer and a minimum of one frame.

Other aggregation methods were also evaluated using the same frame-level pregnancy probabilities. Mean-probability pooling used the average probability across all frames, whereas maximum pooling used only the highest probability. The 10% trimmed mean excluded the lowest 10% and highest 10% of frame probabilities and averaged the remaining values. The number of frames excluded from each end was rounded down to a whole number; for videos with fewer than 10 frames, all probabilities were averaged without exclusion. For each method, a video was classified as pregnant if the resulting score was at least 0.5.

We additionally assessed performance at the individual sow level. To this end, the video-level decisions of each sow were combined, and a sow was classified as pregnant when at least half of its videos were classified as pregnant; ties were therefore classified as pregnant.

### 2.6. Repeated Hold-Out Evaluation

Sows were randomly allocated to training, validation and test sets at a 60:20:20 ratio. This procedure was repeated ten times using different random seeds to assess performance variability across data partitions. The random seeds for split-1 through split-10 were 42, 15, 126, 7, 23, 77, 314, 777, 2024 and 3407, respectively. Test sets could overlap across repetitions; these were repeated hold-out evaluations rather than ten disjoint cross-validation folds. Within each repetition, all frames and videos from a given sow were assigned to the same subset. The same partitions were used for all four experimental conditions to allow direct comparisons. The main Xception analysis used all ten repetitions. Table 4 and Table 5 summarize the average numbers of sows, frames and videos across the ten splits. Performance is reported as the mean ± SD across repeated splits or held-out sessions, as appropriate.

### 2.7. Training and Evaluation

For the repeated hold-out evaluation, training was performed using data from both Datasets A and B, with pregnant-sow data limited to the 18–25-day post-insemination period. Within each of the ten repeated splits, four Xception models were trained under the following conditions: Original, Preprocessing, Mixup and Combined. This resulted in 40 Xception models (10 random splits × 4 conditions). Before model input, the three-channel images were normalized using the ImageNet mean (0.485, 0.456, 0.406) and standard deviation (0.229, 0.224, 0.225). Each model was trained for up to 100 epochs with a batch size of 64, using the Adam optimizer with a learning rate of 1 × 10^−4^ and the cross-entropy loss function. Early stopping was applied if the validation loss did not improve for 5 consecutive epochs. The model checkpoint was selected for each split and condition using the lowest validation loss before evaluation on the held-out test set. Computations were performed using an NVIDIA TITAN RTX GPU and an Intel Xeon W-2133 CPU with PyTorch 2.1.0.

ConvNeXt-Tiny [32] and ViT-Small [33] were included as additional comparison models, initialized with ImageNet-pretrained weights and trained using images resized to 224 × 224 pixels. The optimizer, learning rate, batch size and early-stopping criterion were the same as for Xception. These comparisons used the first three sow-level splits (split-1–split-3) from the main Xception analysis, with the corresponding Xception results used for matched comparisons.

Cross-site evaluation was performed to assess generalization between the two study sites. For A-to-B evaluation, Xception was trained and validated using only the existing Dataset A training and validation sets and evaluated on the corresponding Dataset B test set. The reverse procedure was used for B-to-A evaluation. Both directions were evaluated under all four experimental conditions using split-1 through split-3. Data from the target site were not used for training or checkpoint selection.

Leave-one-session-out (LOSO) evaluation was performed to assess generalization to an unseen acquisition session. LOSO evaluation was conducted separately within each dataset. Each of the six sessions was held out in turn and excluded from both training and validation, while sows from the remaining five sessions were allocated to training and validation sets at an approximately 75:25 ratio. All four experimental conditions were evaluated, yielding 48 separately trained models.

Performance was calculated separately for each held-out session. For sessions containing both pregnant and nonpregnant videos, all metrics were summarized as means ± sample SDs across the same session set within each dataset: A1–A5 for Dataset A (five sessions; 1454 videos) and B1, B2, B4 and B5 for Dataset B (four sessions; 3726 videos). Each session received equal weight. For nonpregnant-only sessions A6, B3 and B6, accuracy and specificity were reported individually and were identical because all reference labels were nonpregnant. Sensitivity, F1-score, Youden’s index and AUC were not reported for these sessions, which were excluded from the mixed-class summaries.

To investigate whether session-associated information was detectable independently of differences in pregnancy-class composition, acquisition-session classification was performed separately for Datasets A and B using nonpregnant videos only. Frozen ImageNet-pretrained Xception features were averaged across sampled frames and videos to obtain one feature vector per sow. A class-weighted logistic regression classifier was evaluated using three-fold stratified group cross-validation at the sow level, with feature standardization fitted exclusively on the training subset of each fold. Performance was calculated from pooled out-of-fold predictions, and significance was assessed using 199 permutations of sow-level session labels within the fixed folds. Detailed methods and results are provided in Supplementary Table S2 and its accompanying text.

Supplementary preprocessing comparisons evaluated wavelet denoising alone, CLAHE alone and their sequential application without Mixup, using the first three sow-level splits and the corresponding Original results.

A conventional-augmentation baseline was evaluated using the first three sow-level splits. Training frames underwent random horizontal flipping with a probability of 0.5, rotation within ±10°, and brightness and contrast adjustments with factors ranging from 0.9 to 1.1. These transformations were applied only during training, without wavelet denoising, CLAHE or Mixup; all other training settings matched Original. Horizontal flipping and rotations within ±10° have been used in ultrasound standard-plane classification [34], and brightness and contrast perturbations have been used in breast-ultrasound classification [35]. The brightness and contrast ranges used here were narrower than those reported in the latter study.

An additional comparison of direct resizing and letterbox resizing was conducted using the first three sow-level splits (split-1–split-3), with the corresponding Original models used as the baseline. Separate Xception models were trained using letterbox-resized inputs, with proportional resizing and centered black padding to 299 × 299 pixels. During testing, the existing masking step was retained, and only the final direct resizing was replaced with letterbox resizing to 299 × 299 pixels.

Each model was evaluated on the held-out test videos specified by its evaluation design, with results reported separately for Datasets A and B, as applicable. Performance was summarized for videos from pregnant sows examined during days 18–21 and days 22–25, as well as for the overall 18–25-day period. For the analyses in Table 6, the same held-out nonpregnant videos were used in each pregnant-sow acquisition-period comparison, irrespective of the availability or value of their acquisition-day information. For the overall 18–25-day results, pregnant videos from the two periods were combined, whereas each nonpregnant video was counted only once.

An exploratory analysis of nonpregnant-video specificity by acquisition period was conducted using the existing video-level predictions from the ten repeated sow-level splits underlying Table 6, without additional model training. Dataset A videos were grouped into days 15–17, days 18–25 and Unknown using filename-recorded days from matched original videos. Dataset B videos were grouped into days 11–17, days 18–25, days 26–29 and Unknown using the reconstructed intervals described in Section 2.1. For each dataset and experimental condition, specificity was calculated separately within each split for each group and for all nonpregnant test videos combined (Overall). Unknown videos were evaluated as a separate group and retained in Overall, but were excluded from groups defined by acquisition day. Group-specific specificity was summarized as the mean ± sample SD across splits containing at least one eligible video; splits without eligible videos were omitted rather than assigned zero specificity. Sample counts and the number of evaluable splits are reported in Table S1, and specificity results are reported in Table S3.

### 2.8. Evaluation Metrics

The evaluation metrics were accuracy, specificity, precision, sensitivity, F1-score, and Youden’s index. Accuracy represents the proportion of correct predictions made by the model. Specificity represents the proportion of nonpregnant videos correctly classified as nonpregnant, whereas precision quantifies the proportion of pregnant videos correctly classified among those predicted to be pregnant. Additionally, sensitivity (or recall) indicates the proportion of actual pregnant videos correctly identified by the model. The F1-score is the harmonic mean of precision and sensitivity and reflects the balance between the two metrics. Youden’s index, which ranges from −1 to 1, is calculated by subtracting 1 from the sum of sensitivity and specificity and indicates combined performance based on both metrics. For consistency with the other metrics in Table 6, Youden’s index was multiplied by 100 and reported on a scale from −100 to 100. Equivalently, when sensitivity and specificity are expressed as percentages, the reported value is calculated as sensitivity (%) + specificity (%) − 100.

Positive predictive value (PPV), equivalent to precision, and negative predictive value (NPV) were additionally summarized for Combined over the overall 18–25-day period. PPV was calculated as TP/(TP + FP), and NPV as TN/(TN + FN), where TP, FP, TN, and FN denote true-positive, false-positive, true-negative, and false-negative video classifications, respectively, with pregnancy treated as the positive class. Both values were multiplied by 100 and are reported as the mean ± SD across the ten repeated splits.

In addition, the area under the receiver operating characteristic (ROC) curve, hereafter the AUC, was computed for each condition from the video-level proportion score—the proportion of frames predicted as pregnant—rather than the binary video-level prediction. The ROC curve was obtained by varying the video-level decision threshold applied to this score, whereas the frame-level threshold of 0.5 was kept fixed. The AUC was computed over days 18–25, with each nonpregnant video included once, as in the corresponding rows of Table 6.

The 95% confidence intervals (CIs) for sow-level sensitivity and specificity under Combined, video-level AUC under each of the four conditions, and the paired differences between Combined and Original in video-level sensitivity, specificity, Youden’s index and video-level AUC were obtained using a cluster bootstrap with 2000 replicates. In each replicate, sows were resampled with replacement within each dataset, with the same resampling weight applied to all videos from a sow across every split in which it appeared. Metrics were recalculated for each split and averaged across the ten splits. For paired comparisons, identical resampling weights were applied to both conditions, and Combined-minus-Original differences were calculated within each replicate. The 2.5th and 97.5th percentiles of the bootstrap estimates defined the 95% CIs. These were marginal percentile intervals without adjustment for multiple comparisons.

## 3. Results and Discussion

Table 6 summarizes video-level performance across ten repeated sow-level splits under Original, Preprocessing, Mixup and Combined. Over days 18–25, Combined yielded modestly higher mean specificity and Youden’s index than Original in both datasets, with numerically similar mean sensitivity. Mean accuracy was also higher with Combined: 84.5% versus 83.9% in Dataset A and 85.3% versus 84.5% in Dataset B. Because the study aimed to support the identification of both nonpregnant and pregnant sows, the following comparisons emphasize sensitivity, specificity and their balance rather than accuracy alone.

Preprocessing alone did not consistently improve classification performance. Mean accuracy was slightly lower than with Original in both datasets. Mean Youden’s index decreased from 61.9 to 60.4 in Dataset A and remained similar in Dataset B (68.3 versus 68.4). Although wavelet denoising and CLAHE were intended to reduce noise and enhance contrast, their sequential application without Mixup did not yield a consistent classification benefit under the evaluated settings.

For each condition in Table 6, the mean positive-class proportions across the ten test splits were 57.5%, 47.3% and 69.2% in Dataset A, and 47.9%, 43.2% and 62.7% in Dataset B, for days 18–21, 22–25 and 18–25, respectively. These proportions refer to videos labeled as pregnant and were calculated separately within each split before averaging. The same nonpregnant test videos were included in each period-specific analysis, whereas the overall 18–25-day analysis combined pregnant videos from both periods and included each nonpregnant video only once. Consequently, the overall analysis had a higher positive-class proportion, and its accuracy is not a weighted average of the two period-specific accuracies.

Across the ten repeated splits, Mixup produced a different sensitivity–specificity balance from Original. It yielded the highest mean specificity among the four conditions in both datasets, increasing specificity from 73.5% with Original to 78.2% in Dataset A and from 83.0% to 90.6% in Dataset B. However, mean sensitivity decreased from 88.4% to 86.4% and from 85.3% to 79.9%, respectively. This reduction was particularly apparent during days 18–21 in Dataset B, where sensitivity decreased from 75.7% to 67.0%. Thus, Mixup improved the identification of nonpregnant videos while increasing the proportion of pregnant videos classified as nonpregnant. This direction was not reproduced in the first three splits of Dataset A: Mixup yielded higher mean sensitivity than Original (92.1% versus 86.3%) but lower mean specificity (70.3% versus 74.2%; Table S4). The ten-split pattern therefore describes an average over the evaluated partitions rather than a consistent direction of change across all subsets of repetitions.

Across the ten repeated splits, adding preprocessing to Mixup shifted the mean performance balance toward greater sensitivity. Over days 18–25, mean sensitivity increased from 86.4% to 88.8% in Dataset A and from 79.9% to 85.2% in Dataset B, whereas specificity decreased from 78.2% to 74.8% and from 90.6% to 85.4%, respectively. Relative to Original, Combined retained modest mean specificity gains with numerically similar overall sensitivity. Its mean Youden’s index was higher than that of Original (63.6 versus 61.9 in Dataset A and 70.6 versus 68.3 in Dataset B), but was lower than that of Mixup in Dataset A (63.6 versus 64.6) and similar in Dataset B (70.6 for both). In the first three splits of Dataset A, however, adding preprocessing to Mixup reduced mean sensitivity from 92.1% to 86.3% and increased specificity from 70.3% to 80.2% (Supplementary Table S4). Thus, the direction of the observed trade-off depended on the set of partitions summarized, and the combination did not provide a uniform improvement across metrics.

Sensitivity was lower during days 18–21 than during days 22–25 under all four conditions in both datasets. For Combined, mean sensitivity was 85.8% versus 93.3% in Dataset A and 75.3% versus 97.2% in Dataset B. Because the same held-out nonpregnant videos were included in each gestational-period analysis, specificity was identical across periods within each dataset and condition. In addition, accuracy, precision and F1-score in the period-specific rows and the overall 18–25-day rows reflect different class proportions: the overall analysis combines pregnant videos from both periods while including each nonpregnant video only once. Lower sensitivity during days 18–21 indicates that a larger proportion of videos from pregnant sows was classified as nonpregnant during this early period. This distinction is important for the intended diagnostic-support role of the system, because improved identification of nonpregnant videos must be considered alongside missed pregnancy detections.

The lower early-period sensitivity is compatible with gestational changes in the visibility of pregnancy-related structures [22,23], but the present comparison does not isolate gestational age as the cause. Each sow contributed study videos from a single time point, and the period groups differed in session composition. In Dataset A, 199 of the 204 pregnant sows examined during days 18–21 came from sessions 1–3 (Table 2).

Compared with the 22–25-day window evaluated by Schwier et al. [20], the present study also included days 18–21 post-insemination. Kim et al. [21] likewise included days 18–21 but evaluated image-based classification, whereas the present study evaluated video-level decisions obtained by aggregating frame predictions. These differences in evaluation windows and units provide context for the present findings but do not establish superior diagnostic performance. Dataset A shares 472 sows and 1428 source videos with Kim et al. [21]. Consequently, comparisons with that study are not comparisons between independent datasets and should not be interpreted as independent replication or external validation.

Sample counts and acquisition-period-specific specificity are presented in Supplementary Tables S1 and S3, respectively. In Dataset A, restricting the analysis to nonpregnant videos with filename-recorded acquisition days of 18–25 yielded mean specificities of 38.9% for Original and 37.8% for Combined, compared with 73.5% and 74.8%, respectively, when all nonpregnant test videos were included. The corresponding values were 35.5% and 32.1% for days 15–17, and 78.9% and 80.9% for Unknown. The 18–25-day test subset contained only 12 distinct records, with 45 video evaluations across eight evaluable splits; the 15–17-day subset contained 11 distinct records, with 72 video evaluations across nine evaluable splits. These small subsets showed substantial between-split variability.

In Dataset B, mean specificity was higher for reconstructed intervals of 18–25 days than for 11–17 days under all four conditions. For Original and Combined, respectively, mean specificity was 84.3% and 85.7% for days 18–25, compared with 78.5% and 82.0% for days 11–17. The corresponding values were 91.9% and 92.2% for days 26–29, and 84.9% and 87.7% for Unknown. Overall specificity was 83.0% for Original and 85.4% for Combined.

In the main Table 6 analysis, which restricted pregnant videos to days 18–25 and included all corresponding nonpregnant test videos, the paired Combined–Original differences in video-level sensitivity and specificity were +0.34 percentage points (95% CI −1.31 to +2.03) and +1.31 percentage points (−1.91 to +4.68), respectively, in Dataset A. The corresponding difference in Youden’s index, expressed on the percentage-point scale, was +1.65 points (−1.92 to +5.32). In Dataset B, the differences were −0.12 percentage points (−1.13 to +0.88) for sensitivity, +2.38 percentage points (+0.74 to +3.96) for specificity and +2.26 points (+0.31 to +4.06) for Youden’s index. Only the Dataset B specificity and Youden’s index intervals excluded zero. These results support a modest improvement at the prespecified operating point in Dataset B under the evaluated conditions, but do not establish improvement across both datasets or all performance measures. The intervals were not adjusted for multiple comparisons.

Table 7 summarizes video-level AUC across ten repeated splits, using the proportion of frames predicted as pregnant as the video-level score. Mean AUC was numerically highest with Mixup in both datasets (0.911 in Dataset A and 0.940 in Dataset B). Combined yielded mean AUCs of 0.902 and 0.934, compared with 0.901 and 0.928 for Original, respectively. The paired AUC difference between Combined and Original was +0.0004 (95% CI −0.0131 to +0.0130) in Dataset A and +0.0052 (95% CI −0.0006 to +0.0109) in Dataset B. Both confidence intervals included zero. These paired comparisons did not establish improved discrimination for Combined relative to Original, although they also do not establish equivalence between the conditions. The modest mean differences in Table 6 describe performance at the prespecified operating point, whereas AUC summarizes ranking by the video-level proportion score across video-level thresholds. Because that score is constructed using a fixed frame-level threshold of 0.5, the AUC analysis does not remove dependence on the frame-level decision threshold.

At the frame level, Combined achieved mean accuracies of 77.2 ± 2.6% in Dataset A and 77.3 ± 0.9% in Dataset B across the ten splits, with corresponding AUCs of 0.842 ± 0.027 and 0.856 ± 0.010. Mixup yielded higher mean frame-level AUCs of 0.848 ± 0.027 and 0.864 ± 0.011, respectively. Frame-level AUC used individual frame probabilities, whereas video-level AUC used the proportion of frames classified as pregnant. Frame labels were inherited from the video-level reference standard; therefore, these metrics describe agreement with video-level pregnancy status rather than independently annotated frame-level diagnostic accuracy. Mean video-level AUC was numerically higher than frame-level AUC for both Mixup and Combined in both datasets. However, frame-level and video-level AUCs concern different evaluation units and score definitions, and frame labels were inherited from video-level pregnancy status. Their difference therefore should not be interpreted as a controlled estimate of the benefit attributable to aggregation.

Performance was also evaluated at the sow level to complement the video-level analysis. Combined achieved a mean specificity of 77.7 ± 12.7% (95% CI 71.9–83.2%) and sensitivity of 91.6 ± 5.3% (95% CI 88.7–94.0%) in Dataset A. The corresponding values in Dataset B were 85.4 ± 9.0% (95% CI 82.4–88.3%) and 88.1 ± 5.4% (95% CI 86.0–90.0%), respectively. With Original, mean specificity and sensitivity were 74.8 ± 14.7% and 91.4 ± 4.2% in Dataset A, and 83.3 ± 11.4% and 87.6 ± 7.3% in Dataset B. Values are means ± SD across the ten repeated splits.

Over days 18–25, Combined yielded video-level PPV and NPV of 89.1 ± 4.3% and 76.3 ± 8.7% in Dataset A, and 91.1 ± 4.2% and 78.2 ± 5.7% in Dataset B, respectively, across the ten repeated splits. The mean proportions of pregnant videos across the ten test splits were 69.2% in Dataset A and 62.7% in Dataset B. These proportions describe the evaluated video samples rather than herd-level pregnancy prevalence, and predictive values may differ in populations with different pregnancy prevalence or sampling characteristics.

Within the evaluated samples, predictions of pregnancy had a higher proportion of correct classifications than predictions of nonpregnancy. The mean NPVs of 76.3% in Dataset A and 78.2% in Dataset B indicate that approximately 24% and 22%, respectively, of video-level nonpregnant predictions were incorrect on average across the ten splits. These predictive values depend on the pregnancy prevalence and sampling characteristics of the evaluated population. This distinction is relevant to the intended role of the system as diagnostic support, particularly when interpreting nonpregnant predictions. Whether providing these predictions improves specialist assessment or reproductive management remains to be evaluated.

Alternative aggregation rules did not provide a consistent advantage over the predefined majority-voting rule. Maximum and top-percentage pooling increased sensitivity at the cost of substantially lower specificity, whereas the trimmed mean and majority voting yielded similar mean Youden’s indices (Supplementary Table S5).

The additional-backbone comparisons showed configuration-dependent effects: Combined yielded higher mean accuracy and Youden’s index than Original for Xception and ConvNeXt-Tiny in both datasets, but these benefits were not reproduced with ViT-Small. These descriptive comparisons used the first three shared sow-level splits without architecture-specific optimization and do not establish statistical superiority (Supplementary Table S6).

Table 8 summarizes cross-site performance using the first three repeated splits. The effects of preprocessing and Mixup differed between transfer directions. For A-to-B evaluation, Preprocessing yielded the highest mean accuracy and Youden’s index. Compared with Original, specificity increased from 47.4% to 63.5%, whereas sensitivity decreased from 85.4% to 78.4%. This benefit in mean Youden’s index was not reproduced in B-to-A evaluation, where Preprocessing and Original yielded similar values (37.3 and 37.6).

For B-to-A evaluation, Mixup yielded the highest mean specificity and Youden’s index, whereas Combined yielded the highest mean accuracy and sensitivity. Compared with Mixup, Combined increased sensitivity from 69.5% to 79.2% but reduced specificity from 80.2% to 61.5%. Combined did not yield the highest mean Youden’s index in either transfer direction. Substantial variability was also observed across splits, particularly for A-to-B specificity under Combined (50.6 ± 30.2%). These findings indicate that the performance balance observed with a given training condition was not consistently maintained across sites.

Under Combined, training on both datasets yielded higher mean accuracy on the corresponding held-out test sets than training on the other site alone: 84.5% versus 73.8% in Dataset A and 84.5% versus 72.0% in Dataset B across the same three splits. Joint training was associated with higher accuracy in both datasets, although this comparison did not separate the effects of increased training-data volume from those of including target-site data.

Table 9 reports LOSO performance separately for sessions containing both classes and nonpregnant-only sessions. Across the mixed-class sessions, Original yielded mean specificities of 41.1% in Dataset A and 77.4% in Dataset B, compared with 73.5% and 83.0%, respectively, in the ten repeated random splits. These comparisons are descriptive because the LOSO summaries give equal weight to mixed-class held-out sessions, whereas the random-split results average performance across repeated test sets drawn from all sessions. Differences in training composition and evaluated populations also limit direct comparison. The findings are compatible with sensitivity to acquisition-session characteristics but do not establish reliance on session-specific cues or data leakage.

Among mixed-class sessions in Dataset A, Mixup yielded the highest mean accuracy, specificity and Youden’s index. Compared with Original, mean specificity increased from 41.1% to 64.0%, whereas sensitivity decreased from 90.1% to 81.3%. Combined yielded intermediate mean sensitivity and specificity of 83.7% and 55.7%, respectively. In Dataset B, Combined yielded the highest mean accuracy, specificity and Youden’s index, although its specificity and Youden’s index were close to those of Original (77.5% versus 77.4% and 56.8 versus 56.1, respectively), and its mean AUC was slightly lower (0.864 versus 0.867). No condition consistently performed best across both datasets.

Results also varied across the nonpregnant-only sessions, where accuracy and specificity were identical. Preprocessing yielded the highest specificity in A6 (80.0%), Combined in B3 (73.8%) and Original in B6 (64.7%). These individual-session results further illustrate variation across acquisition settings and are reported separately from the mixed-class summaries.

In the supplementary session-classification analysis, balanced accuracy was 30.4% for Dataset A and 54.4% for Dataset B, exceeding the six-session chance level of 16.7% (permutation *p* = 0.005 for each dataset; Supplementary Table S2). These results demonstrate that session-associated information remained detectable in the masked nonpregnant ultrasound images. However, they do not establish that the pregnancy classifiers relied on this information or quantify its contribution to pregnancy-classification performance.

The effects of wavelet denoising and CLAHE differed between datasets. Without Mixup, their sequential application did not yield higher mean accuracy, Youden’s index or AUC than either component alone in either dataset (Supplementary Table S7).

Compared with Original, conventional augmentation increased mean sensitivity but reduced specificity in both datasets. It did not provide a consistent advantage across datasets or performance metrics (Supplementary Table S4).

Over the overall 18–25-day period, letterbox resizing yielded numerically higher mean sensitivity but lower mean specificity and AUC than the corresponding Original Xception baseline across the first three shared sow-level splits in both datasets (Supplementary Tables S6 and S8). Letterbox resizing yielded no consistent improvements across the evaluated metrics under the tested experimental conditions.

Selected Grad-CAM [36] cases are presented in Supplementary Figure S1 as qualitative examples. However, these visualizations do not serve as definitive evidence for confirming anatomical localization, explaining classification errors, or identifying the underlying causes of performance differences across conditions.

To provide preliminary context for computational feasibility, a benchmark was conducted using six videos. Xception processed a 10 s video containing 99 frames in approximately 2.2 s on the TITAN RTX GPU and 8.3 s on the Xeon W-2133 CPU using six threads. Timing included decoding, preprocessing, inference and vote aggregation.

Taken together, the four-condition comparison indicates performance trade-offs, with modest benefits of Combined over Original at the prespecified operating point. Across the ten repeated splits, Combined yielded higher mean accuracy, specificity and Youden’s index in both datasets, with numerically similar mean sensitivity. The paired video-level comparisons provided evidence of higher specificity and Youden’s index in Dataset B, where the corresponding marginal 95% confidence intervals excluded zero, although no adjustment was made for multiple comparisons. The corresponding intervals in Dataset A included zero. Compared with Mixup alone, Combined yielded higher mean sensitivity and lower mean specificity in the ten-split analysis, making the balance between missed pregnancies and false-positive predictions relevant to the choice of configuration. This trade-off varied with the partitions examined, and the paired AUC comparisons did not establish improved discrimination relative to Original. The evidence therefore supports a limited benefit at the evaluated operating point, rather than superiority across metrics or acquisition settings.

The interventions also act at different stages of the workflow: wavelet denoising and CLAHE transform the images used for training and evaluation, whereas Mixup interpolates training images and labels. A possible explanation for some condition-dependent differences is that these interventions changed the distribution of frame probabilities relative to the fixed threshold of 0.5, which in turn changed the video-level proportion scores. This hypothesis was not tested directly, and neither the direction of the trade-off nor improved calibration follows from the use of Mixup alone. Comparing frame-probability distributions and calibration across conditions could help assess this explanation. The present results also do not establish whether preprocessing preserved, removed or emphasized image information relevant to pregnancy classification.

This study has several limitations. Pregnancy status was established through serial follow-up ultrasonography during routine farm management. The available records did not establish the timing of pregnancy-status confirmation relative to study-video acquisition for each sow. Although the acquisition personnel and the confirming specialist were different, blinding of the specialist to the study videos or initial pregnancy assessments could not be confirmed. The independence of the reference assessment from this information therefore remains uncertain. Early embryonic loss was not assessed in this study, and its occurrence could not be determined from the study videos and the information available to the research team. Individual farrowing records were not available to the research team for independent verification of the pregnancy-status labels. Because acquisition windows differed between pregnant and nonpregnant sows, the reported specificity should not be interpreted as an estimate from a strictly day-matched cohort.

A matched reader study comparing specialist performance with and without CADx assistance on the same videos was not conducted. Therefore, the present results do not establish whether CADx assistance improves specialist diagnostic performance or reproductive management. Such an evaluation is needed to determine the clinical added value of the system.

The data were collected at two study sites using the same acquisition-device model and experienced operators. Cross-site and LOSO evaluations extended the assessment beyond random splits, but generalizability to independently collected data from other farms, devices and operators remains to be established. Equal-session averaging gave small and large sessions the same weight, although estimates from smaller sessions may be less precise. The above-chance session-classification performance supports concern about session-related confounding, particularly because pregnancy-class proportions differed across sessions. Restricting this analysis to nonpregnant sows removed pregnancy-class variation from the classification task, but did not distinguish acquisition-related differences from biological differences among the animals sampled in each session. The extent to which these session-associated differences influenced pregnancy classification therefore remains to be determined. The supplementary comparisons in Tables S4–S8 used three shared splits, whereas the acquisition-period analysis in Tables S1 and S3 used the ten splits underlying Table 6.

Bootstrap CIs quantified sampling uncertainty conditional on the fitted models and existing partitions and did not include additional model retraining. The paired comparisons used marginal percentile CIs without adjustment for multiple comparisons. The positive specificity and Youden’s index differences in Dataset B should therefore be interpreted within this scope and do not establish a general advantage across datasets or metrics. Intervals that include zero do not establish equivalence or non-inferiority. Between-split SDs for individual conditions describe variation across partitions and should not be interpreted as uncertainty intervals for the paired differences.

Frame-level labels were inherited from video-level pregnancy status, so non-diagnostic frames received the same label as diagnostic frames without independent frame-level annotation. In Dataset A sessions 1–5, pregnant-sow training frames were additionally selected for visible gestational sacs by personnel who knew the pregnancy labels. This selection may have enriched the pregnant training samples for clearly visible diagnostic features and introduced selection bias. Its selective application also created differences in training-data preparation across classes, sessions and datasets, which could have influenced performance. Although video-level testing used unfiltered videos, the effect of this training-frame selection was not separately evaluated.

The findings also concern the specific preprocessing settings evaluated here: the Haar-based thresholding procedure was not compared with alternative speckle-specific approaches [10,11], and the CLAHE parameters were not optimized for these data. Because preprocessing was applied during both training and evaluation, its effects on model learning could not be isolated from those of changing the evaluation inputs. Alternative aggregation rules were examined, but multiple-instance and temporal video models were not evaluated.

## 4. Conclusions

This study identified configuration-dependent trade-offs in sow pregnancy classification using low-frequency portable ultrasound videos, Xception-based frame classification and video-level aggregation. Across ten repeated sow-level splits, Combined yielded numerically higher mean accuracy, specificity and Youden’s index than Original in both datasets, with numerically similar mean sensitivity. However, paired 95% confidence intervals for the specificity and Youden’s index differences excluded zero only in Dataset B, without adjustment for multiple comparisons. These findings support a limited, dataset-dependent benefit at the evaluated operating point.

The observed benefits do not establish uniform superiority. Paired AUC comparisons did not establish improved discrimination, and Mixup yielded the highest mean specificity and AUC in the ten-split analysis. Combined yielded higher mean sensitivity than Mixup, but the direction of this trade-off varied with the partitions examined. Supplementary comparisons and cross-site and LOSO evaluations further showed that performance depended on the evaluated configuration and acquisition setting.

Future work should prioritize a more diverse dataset spanning farms, acquisition sessions, devices and operators, with both pregnancy classes and relevant gestational stages represented across acquisition settings where feasible. Recording acquisition metadata and prespecifying evaluations on held-out farms and sessions would help determine which session-associated differences influence pregnancy classification and whether their effects persist in independently collected data. Methodological work should compare regularization-based augmentation, including Mixup and alternative image-mixing strategies such as CutMix and FMix, with ultrasound-oriented denoising, evaluating their effects separately and jointly under matched data partitions and controlled input processing. These comparisons should assess whether benefits persist across acquisition settings and should include paired uncertainty estimates, calibration and early-gestation sensitivity, rather than relying on mean accuracy alone. Independent evaluation is needed to assess whether the observed benefits of Combined extend to other acquisition settings. A further priority is a matched reader study comparing specialist performance with and without CADx assistance on the same videos to determine the clinical added value of the system.

## Figures and Tables

**Table 1:** Specifications of the ultrasound equipment used for data collection.

Item	Specification
Manufacturer	Songkang GLC Co. Seongnam, Republic of Korea
Model	SV-1
Probe	Abdominal Sector Probe
Scan Angle	Sector scan, 80°
Frequency	3.5 MHz
Penetration Depth	100–180 mm
Display Mode	B-Mode
Gray Scale	256 Levels
Video Resolution	1120 × 624 pixels
Frame Rate	10 fps

**Table 2:** Details of the dataset collected at the College of Animal Life Sciences, Kangwon National University, Republic of Korea.

Dataset A	Nonpregnant		Pregnant (18–21)		Pregnant (22–25)		All	
	Head	Frame	Head	Frame	Head	Frame	Head	Frame
1	15	533	39	1544	47	2051	101	4128
2	12	508	99	3974	8	465	119	4947
3	17	861	61	3083	20	1136	98	5080
4	36	1280	3	141	54	2772	93	4193
5	61	2610	2	92	7	265	70	2967
6	11	580	0	0	0	0	11	580
Total	152	6372	204	8834	136	6689	492	21,895

**Table 3:** Details of the dataset collected at the National Institute of Animal Science, Republic of Korea.

Dataset B	Nonpregnant		Pregnant (18–21)		Pregnant (22–25)		All	
	Head	Frame	Head	Frame	Head	Frame	Head	Frame
1	110	4499	96	3859	91	3660	297	12,018
2	77	4659	110	6580	35	2100	222	13,339
3	13	840	0	0	0	0	13	840
4	15	900	79	4760	82	4920	176	10,580
5	250	15,019	194	11,776	195	11,780	639	38,575
6	56	3399	0	0	0	0	56	3399
Total	521	29,316	479	26,975	403	22,460	1403	78,751

**Table 4:** Average numbers of sows and frames in the training and validation sets used for the ten repeated random splits.

Dataset		Nonpregnant		Pregnant (18–21)		Pregnant (22–25)		All	
		Head	Frame	Head	Frame	Head	Frame	Head	Frame
Dataset A	Train	91	3821	122	5362	81	3987	294	13,170
	Valid	30	1230	40	1714	27	1308	97	4252
Dataset B	Train	311	17,490	286	16,109	241	13,444	838	47,043
	Valid	103	5795	95	5313	80	4436	278	15,544
Total		535	28,336	543	28,498	429	23,175	1507	80,009

**Table 5:** Average numbers of sows and videos in test sets for the ten repeated random splits.

Dataset		Nonpregnant		Pregnant (18–21)		Pregnant (22–25)		All	
		Head	Video	Head	Video	Head	Video	Head	Video
Dataset A	Test	31	94	42	127	28	84	101	305
Dataset B	Test	107	302	99	278	82	229	288	809
Total		138	396	141	405	110	313	389	1114

Values in Table 4 and Table 5 are averages across the ten repeated splits and were rounded to whole numbers. Because totals were calculated using rounded category values, they may differ slightly from those calculated using unrounded averages.

**Table 6:** Video-level performance of Xception under the four experimental conditions across ten repeated sow-level splits.

Method	Dataset	Period (Days)	Accuracy	Specificity	Precision	Sensitivity	F1-Score	Youden’s Index
Original	A	18–21	80.3 ± 3.0	73.5 ± 13.3	82.2 ± 6.7	85.3 ± 7.8	83.3 ± 2.4	58.8 ± 7.8
		22–25	82.8 ± 5.4	73.5 ± 13.3	77.2 ± 8.6	93.1 ± 5.1	83.9 ± 3.9	66.6 ± 10.0
		18–25	83.9 ± 1.6	73.5 ± 13.3	88.7 ± 4.6	88.4 ± 6.0	88.3 ± 1.3	61.9 ± 8.0
	B	18–21	79.5 ± 2.4	83.0 ± 11.1	81.9 ± 7.4	75.7 ± 12.0	77.7 ± 3.7	58.7 ± 5.0
		22–25	89.1 ± 5.7	83.0 ± 11.1	82.4 ± 8.9	97.0 ± 2.4	88.8 ± 4.9	80.1 ± 9.9
		18–25	84.5 ± 2.0	83.0 ± 11.1	90.0 ± 5.0	85.3 ± 7.4	87.2 ± 2.1	68.3 ± 5.4
Preprocessing	A	18–21	79.7 ± 5.9	71.7 ± 15.4	81.2 ± 7.7	85.7 ± 6.3	83.1 ± 4.3	57.4 ± 13.9
		22–25	81.9 ± 6.6	71.7 ± 15.4	76.1 ± 8.9	93.3 ± 5.6	83.3 ± 4.7	64.9 ± 12.4
		18–25	83.5 ± 3.9	71.7 ± 15.4	88.0 ± 5.3	88.7 ± 5.2	88.2 ± 2.6	60.4 ± 13.0
	B	18–21	79.3 ± 3.3	83.8 ± 10.4	82.2 ± 7.1	74.5 ± 11.7	77.3 ± 4.7	58.3 ± 6.7
		22–25	89.5 ± 5.3	83.8 ± 10.4	82.9 ± 8.2	96.9 ± 2.1	89.1 ± 4.5	80.7 ± 9.0
		18–25	84.3 ± 2.6	83.8 ± 10.4	90.3 ± 4.7	84.6 ± 7.3	87.0 ± 2.6	68.4 ± 6.1
Mixup	A	18–21	81.1 ± 3.8	78.2 ± 10.0	84.4 ± 6.0	83.3 ± 7.6	83.4 ± 3.4	61.4 ± 8.0
		22–25	84.4 ± 4.4	78.2 ± 10.0	79.8 ± 7.6	91.2 ± 4.6	84.8 ± 3.5	69.4 ± 8.4
		18–25	83.9 ± 3.2	78.2 ± 10.0	90.2 ± 3.9	86.4 ± 6.0	88.1 ± 2.5	64.6 ± 7.6
	B	18–21	79.3 ± 3.3	90.6 ± 7.4	88.4 ± 6.7	67.0 ± 13.6	74.9 ± 6.4	57.6 ± 7.2
		22–25	92.8 ± 3.5	90.6 ± 7.4	89.3 ± 7.6	95.7 ± 2.6	92.1 ± 3.4	86.3 ± 5.9
		18–25	83.9 ± 2.9	90.6 ± 7.4	94.0 ± 4.1	79.9 ± 8.4	86.0 ± 3.3	70.6 ± 3.1
Combined	A	18–21	81.1 ± 4.6 (0.8▲)	74.8 ± 12.6 (1.3▲)	82.8 ± 6.0 (0.6▲)	85.8 ± 9.0 (0.5▲)	83.8 ± 4.3 (0.6▲)	60.6 ± 10.0 (1.8▲)
		22–25	83.5 ± 5.8 (0.7▲)	74.8 ± 12.6 (1.3▲)	77.8 ± 7.9 (0.6▲)	93.3 ± 3.3 (0.1▲)	84.5 ± 4.4 (0.6▲)	68.0 ± 11.0 (1.4▲)
		18–25	84.5 ± 3.2 (0.6▲)	74.8 ± 12.6 (1.3▲)	89.1 ± 4.3 (0.4▲)	88.8 ± 6.0 (0.3▲)	88.7 ± 2.5 (0.4▲)	63.6 ± 9.5 (1.7▲)
	B	18–21	80.5 ± 1.5 (1.0▲)	85.4 ± 8.2 (2.4▲)	83.7 ± 6.5 (1.8▲)	75.3 ± 10.2 (0.4▼)	78.5 ± 3.1 (0.8▲)	60.7 ± 3.3 (2.0▲)
		22–25	90.5 ± 4.6 (1.4▲)	85.4 ± 8.2 (2.4▲)	84.1 ± 7.7 (1.8▲)	97.2 ± 1.0 (0.2▲)	90.0 ± 4.4 (1.2▲)	82.7 ± 8.2 (2.6▲)
		18–25	85.3 ± 1.1 (0.8▲)	85.4 ± 8.2 (2.4▲)	91.1 ± 4.2 (1.1▲)	85.2 ± 5.7 (0.1▼)	87.8 ± 1.4 (0.6▲)	70.6 ± 3.3 (2.3▲)

Values are means ± standard deviations across ten repeated splits. Parenthetical values show differences from Original in percentage points for percentage-based metrics and in points for Youden’s index. Differences were calculated from unrounded means and then rounded to one decimal place; therefore, they may differ slightly from differences calculated directly from the displayed means. ▲ and ▼ indicate numerical increases and decreases, respectively, and do not denote statistical significance.

**Table 7:** Video-level AUC for the four experimental conditions.

Dataset	Condition	Video AUC (Mean ± SD Across Splits)	95% CI
Dataset A	Original	0.901 ± 0.024	0.876–0.925
	Preprocessing	0.895 ± 0.039	0.867–0.918
	Mixup	0.911 ± 0.030	0.886–0.931
	Combined	0.902 ± 0.032	0.876–0.924
Dataset B	Original	0.928 ± 0.015	0.916–0.941
	Preprocessing	0.928 ± 0.019	0.915–0.940
	Mixup	0.940 ± 0.008	0.930–0.951
	Combined	0.934 ± 0.008	0.922–0.945

Values are the mean ± standard deviation (SD) of the video-level AUC across the ten repeated splits; 95% confidence intervals were obtained with a sow-level cluster bootstrap (2000 replicates) in which the same sows were resampled jointly across the ten splits.

**Table 8:** Cross-site video-level performance of Xception over days 18–25 using the first three repeated splits.

Direction	Condition	Accuracy	Sensitivity	Specificity	F1-Score	Youden’s Index
A → B	Original	71.2 ± 2.2	85.4 ± 3.3	47.4 ± 9.0	78.8 ± 1.1	32.7 ± 6.9
	Preprocessing	72.7 ± 0.9	78.4 ± 9.5	63.5 ± 18.3	78.1 ± 1.5	41.8 ± 8.8
	Mixup	71.6 ± 2.4	79.5 ± 9.6	58.6 ± 22.1	77.8 ± 0.8	38.0 ± 12.6
	Combined	72.0 ± 4.7	84.8 ± 10.8	50.6 ± 30.2	79.2 ± 0.9	35.5 ± 19.4
B → A	Original	69.2 ± 4.6	69.8 ± 3.1	67.8 ± 11.3	75.9 ± 3.3	37.6 ± 12.6
	Preprocessing	65.9 ± 9.6	61.6 ± 17.6	75.7 ± 10.4	70.4 ± 12.6	37.3 ± 10.2
	Mixup	72.8 ± 1.2	69.5 ± 4.7	80.2 ± 6.4	77.9 ± 2.1	49.6 ± 1.7
	Combined	73.8 ± 2.7	79.2 ± 4.2	61.5 ± 5.9	80.7 ± 2.3	40.7 ± 5.7

Values are mean ± standard deviation (SD) across the first three repeated splits. A → B denotes training and validation using Dataset A and testing using Dataset B; B → A denotes the reverse direction. Accuracy, sensitivity, specificity and F1-score are expressed as percentages. Youden’s index is reported on a scale from −100 to 100.

**Table 9:** Video-level performance of Xception in leave-one-session-out evaluation, reported separately for sessions containing both classes and nonpregnant-only sessions.

(a) Sessions containing both classes							
Dataset	Condition	Accuracy	Sensitivity	Specificity	F1-Score	Youden’s Index	AUC
A	Original	69.5 ± 24.6	90.1 ± 3.7	41.1 ± 20.2	74.7 ± 28.5	31.2 ± 18.2	0.771 ± 0.148
	Preprocessing	68.5 ± 26.4	90.1 ± 4.2	40.6 ± 28.7	74.5 ± 28.9	30.7 ± 25.8	0.752 ± 0.166
	Mixup	74.8 ± 11.8	81.3 ± 1.7	64.0 ± 25.6	75.3 ± 24.9	45.4 ± 26.9	0.790 ± 0.170
	Combined	73.0 ± 15.9	83.7 ± 6.3	55.7 ± 22.4	74.7 ± 26.3	39.5 ± 26.1	0.789 ± 0.153
B	Original	76.7 ± 3.4	78.7 ± 6.7	77.4 ± 18.5	82.3 ± 3.3	56.1 ± 14.4	0.867 ± 0.060
	Preprocessing	74.1 ± 10.0	85.3 ± 17.2	51.7 ± 23.2	81.0 ± 9.9	37.0 ± 15.3	0.846 ± 0.072
	Mixup	76.4 ± 12.8	81.7 ± 19.2	66.7 ± 31.0	81.4 ± 12.0	48.4 ± 25.6	0.850 ± 0.084
	Combined	77.6 ± 6.8	79.3 ± 12.1	77.5 ± 22.7	82.5 ± 7.1	56.8 ± 18.7	0.864 ± 0.072
(b) Nonpregnant-only sessions: accuracy = specificity (%)							
Condition		Dataset A6 (*n* = 30 videos)		Dataset B3 (*n* = 42 videos)		Dataset B6 (*n* = 170 videos)	
Original		70.0		19.0		64.7	
Preprocessing		80.0		45.2		42.4	
Mixup		50.0		66.7		20.0	
Combined		40.0		73.8		31.2	

Panel (a): means ± sample SDs across mixed-class held-out sessions, with equal session weights (Dataset A: A1–A5; Dataset B: B1, B2, B4 and B5). Panel (b): individual-session estimates; accuracy equals specificity. Accuracy, sensitivity, specificity and F1-score are expressed as percentages; Youden’s index is reported on a scale from −100 to 100.

## Data Availability

The data presented in this study are available from the corresponding author upon reasonable request.

## Supplementary Materials

The following supporting information can be downloaded at: https://www.mdpi.com/article/10.3390/ani16213470/s1, Table S1: Numbers of nonpregnant records and videos by acquisition period in the complete datasets and repeated test sets; Table S2: Acquisition-session classification using frozen ImageNet features from nonpregnant ultrasound videos; Table S3: Video-level specificity by acquisition-period group using the ten repeated sow-level splits; Table S4: Video-level comparison of conventional augmentation, Mixup and Combined over days 18–25 using the first three repeated splits; Table S5: Comparison of video-level aggregation methods for Combined over days 18–25 using the first three repeated splits; Table S6: Video-level performance of three backbones under four experimental conditions over days 18–25 using the first three repeated sow-level splits; Table S7: Video-level comparison of wavelet denoising and CLAHE without Mixup over days 18–25 using the first three repeated splits; Table S8: Video-level performance of Xception with letterbox resizing over days 18–25 using the first three repeated sow-level splits; Figure S1: Grad-CAM maps for selected true-positive, true-negative, false-positive and false-negative videos at frame index 50. Reference [36] is cited in the Supplementary Materials.
