# Supplementary material for: Computer-Aided Diagnosis System for Sow Pregnancy Using Low-Frequency Portable Ultrasound Videos with Preprocessing and Augmentation Techniques
Source: Animals (Basel). 2026 Nov 3;16(21):3470. doi: 10.3390/ani16213470 (PMC13762119; doi:10.3390/ani16213470)
Supplement: Supplementary file 1 [file animals-16-03470-s001.zip › animals-4611030-supplementary.pdf]

Supplementary Table S1

Nonpregnant records were grouped by available acquisition-day information. Dataset A groups were based on days recorded in the filenames of matched original videos, whereas Dataset B groups were based on intervals reconstructed by linking acquisition records to insemination records. The reconstructed intervals in Dataset B ranged from 11 to 29 days and were not reassigned to the boundaries of the previously stated collection window. Records without usable day information were classified as Unknown. Table S1 summarizes sample availability, and Table S3 reports specificity using the existing predictions from the ten repeated sow-level splits underlying Table 6.

Table S1. Numbers of nonpregnant records and videos by acquisition period in the complete datasets and repeated test sets.

Dataset	Acquisition period (days)	Total records	Total videos	Unique test records	Total video evaluations	Splits included
A	Overall	152	459	135	939	10
	15–17 days	12	36	11	72	9
	18–25 days	15	45	12	45	8
	Unknown	125	378	112	822	10
B	Overall	521	1,466	468	3,016	10
	11–17 days	144	413	129	887	10
	18–25 days	234	626	211	1,271	10
	26–29 days	31	90	24	151	10
	Unknown	112	337	104	707	10

Total records and total videos refer to the complete dataset. Unique test records are distinct records included in at least one of the ten test splits. Total video evaluations count each appearance of a video in a test split; the same video may therefore be counted more than once. Splits included denotes the number of splits containing at least one eligible nonpregnant test video.

Supplementary Table S2

To assess whether the ultrasound images contained information associated with acquisition session, we performed session classification separately for Datasets A and B using nonpregnant videos only. Five evenly spaced frames were sampled from each video, resized to 1120 × 624 pixels, masked, and subsequently resized to 299 × 299 pixels. A frozen ImageNet-pretrained Xception network was used to extract 2048-dimensional features. Features were averaged first across frames within each video and then across videos belonging to the same sow. A class-weighted logistic regression classifier (C = 1) was evaluated using three-fold stratified group cross-validation, with no sow shared between the training and evaluation subsets. Feature standardization was fitted exclusively on the training subset of each fold. Statistical significance was assessed using 199 permutations of sow-level session labels within the fixed folds, with the standardization and classification pipeline refitted for each permutation.

Table S2. Acquisition-session classification using frozen ImageNet features from nonpregnant ultrasound videos.

Dataset	Nonpregnant sows, n	Videos, n	Sessions, n	Balanced Accuracy (%)	Macro-F1	Permutation p-value
A	152	459	6	30.4	0.315	0.005
B	521	1,466	6	54.4	0.555	0.005

Performance was calculated from pooled out-of-fold predictions obtained using three-fold stratified group cross-validation at the sow level. Each sow contributed one feature vector and one out-of-fold prediction. The chance-level balanced accuracy for six-session classification was 16.7%. Permutation p-values were calculated using 199 sow-level label permutations within the fixed cross-validation folds, preserving the session counts in each fold. Feature standardization and classifier fitting were repeated for every permutation.

Session classification achieved balanced accuracies of 30.4% for Dataset A and 54.4% for Dataset B, compared with a chance level of 16.7% for six sessions (Table S2). Both results exceeded all 199 permuted-label results (permutation $p = 0.005$ for each dataset). These findings indicate that session-associated information was detectable in the masked nonpregnant ultrasound images. However, this analysis does not establish that the pregnancy classifiers relied on this information or quantify its contribution to pregnancy-classification performance.

Supplementary Table S3

Table S3. Video-level specificity by acquisition-period group using the ten repeated sow-level splits.

(a) Dataset A

Condition	Overall	15–17 days	18–25 days	Unknown
Original	73.5 ± 13.3	35.5 ± 36.2	38.9 ± 34.2	78.9 ± 11.3
Preprocessing	71.7 ± 15.4	32.7 ± 34.5	30.6 ± 36.5	77.5 ± 13.6
Mixup	78.2 ± 10.0	54.0 ± 22.3	31.3 ± 31.4	83.3 ± 9.5
Combined	74.8 ± 12.6	32.1 ± 29.8	37.8 ± 37.9	80.9 ± 13.8

(b) Dataset B

Condition	Overall	11–17 days	18–25 days	26–29 days	Unknown
Original	83.0 ± 11.1	78.5 ± 13.4	84.3 ± 9.3	91.9 ± 17.2	84.9 ± 14.2
Preprocessing	83.8 ± 10.4	79.5 ± 15.8	85.3 ± 7.2	87.9 ± 19.6	85.2 ± 11.0
Mixup	90.6 ± 7.4	88.0 ± 8.4	91.4 ± 6.5	94.9 ± 10.6	91.6 ± 9.1
Combined	85.4 ± 8.2	82.0 ± 11.2	85.7 ± 7.2	92.2 ± 9.0	87.7 ± 11.2

Values are specificity percentages, expressed as means ± sample standard deviations across evaluable splits. Specificity was calculated separately within each split. Splits without eligible videos were excluded rather than assigned zero specificity. Dataset A summaries use nine splits for days 15–17, eight for days 18–25, and ten for Overall and Unknown; all Dataset B summaries use ten splits. Overall includes all nonpregnant test videos, including Unknown records. Acquisition-period definitions and sample counts are provided in Table S1. Comparisons are descriptive and do not isolate acquisition-period effects from differences in sample and session composition.

In Dataset A, specificity was lower in the subsets with available acquisition-day information than in the Unknown group under all four conditions, but the dated subsets were small and varied substantially across splits. In Dataset B, mean specificity was higher for days 18–25 than for days 11–17 under all four conditions.

Supplementary Table S4

Table S4 compares conventional augmentation with Original, Mixup and Combined using the first three repeated splits. Compared with Original, conventional augmentation increased mean sensitivity but reduced specificity in both datasets. In Dataset A, sensitivity increased from 86.3% to 91.7%, while specificity decreased from 74.2% to 54.7%. Mean accuracy, Youden's index and AUC were also lower than with Original.

In Dataset B, conventional augmentation yielded higher mean accuracy and AUC than Original, although specificity decreased from 81.2% to 77.7%. Its mean accuracy was the highest among the compared conditions, but its mean Youden's index and AUC were lower than those of Mixup and Combined. Conventional augmentation therefore shifted the sensitivity–specificity balance differently from Mixup and did not provide a consistent advantage across datasets or metrics.

Table S4. Video-level comparison of conventional augmentation, Mixup and Combined over days 18–25 using the first three repeated splits.

Dataset	Condition	Accuracy	Sensitivity	Specificity	F1-score	Youden's index	AUC
A	Original	82.6 ± 1.7	86.3 ± 3.5	74.2 ± 12.1	87.3 ± 0.8	60.5 ± 8.9	0.899 ± 0.024
	Conventional augmentation	80.3 ± 1.0	91.7 ± 1.8	54.7 ± 4.2	86.6 ± 0.6	46.4 ± 3.4	0.876 ± 0.031
	Mixup	85.4 ± 4.0	92.1 ± 4.8	70.3 ± 5.7	89.7 ± 3.0	62.4 ± 8.1	0.912 ± 0.034
	Combined	84.5 ± 3.2	86.3 ± 2.6	80.2 ± 6.1	88.5 ± 2.3	66.5 ± 7.8	0.902 ± 0.021
B	Original	83.0 ± 2.3	84.1 ± 10.3	81.2 ± 11.2	86.0 ± 3.0	65.3 ± 1.6	0.919 ± 0.017
	Conventional augmentation	84.9 ± 2.2	89.3 ± 1.2	77.7 ± 4.8	88.1 ± 1.7	66.9 ± 5.3	0.925 ± 0.010
	Mixup	84.6 ± 3.5	83.9 ± 9.9	86.0 ± 9.8	87.1 ± 3.8	69.9 ± 4.7	0.937 ± 0.010
	Combined	84.5 ± 0.6	84.3 ± 5.5	85.1 ± 7.7	87.2 ± 1.1	69.3 ± 2.4	0.928 ± 0.004

Values are mean ± SD across the first three repeated splits. Conventional augmentation comprised random horizontal flipping, rotation, and brightness and contrast adjustments without wavelet denoising, CLAHE or Mixup. Accuracy, sensitivity, specificity and F1-score are expressed as percentages. Youden's index is reported on a scale from −100 to 100.

Supplementary Table S5

Table S5 compares alternative aggregation rules for Combined over days 18–25 using the same frame-level predictions from the first three splits. Maximum pooling and top-percentage pooling yielded high mean sensitivity but substantially lower specificity than majority voting in both datasets. Majority voting, mean-probability pooling and the 10% trimmed mean yielded higher mean Youden's index values than these approaches. The trimmed mean and majority voting produced similar mean Youden's index values: 66.6 versus 66.5 in Dataset A and 69.1 versus 69.3 in Dataset B. These results show that emphasizing the highest frame probabilities increased the detection of pregnant videos at the cost of more false-positive classifications and did not provide a consistent advantage over the predefined majority-voting rule.

At the fixed threshold of 0.5, maximum pooling classified a video as pregnant whenever at least one frame exceeded or equaled that threshold. Its mean specificity of 4.6% in Dataset A and 5.7% in Dataset B therefore indicates that nearly all evaluated nonpregnant videos contained at least one positive frame prediction. Majority voting instead required positive predictions in at least half of the frames. The similar mean Youden's indices obtained with majority voting, mean-probability pooling and the trimmed mean describe similar aggregate performance, but do not establish that these methods classified the same individual videos identically. These comparisons also do not establish their relative performance after method-specific threshold selection.

Table S5. Comparison of video-level aggregation methods for Combined over days 18–25 using the first three repeated splits.

Aggregation method	A Sensitivity	A Specificity	A Youden's index	B Sensitivity	B Specificity	B Youden's index
**Majority voting**	86.3 ± 2.6	80.2 ± 6.1	66.5 ± 7.8	84.3 ± 5.5	85.1 ± 7.7	69.3 ± 2.4
**Mean-probability pooling**	87.6 ± 3.9	78.4 ± 6.6	66.0 ± 9.8	84.8 ± 5.5	84.5 ± 8.0	69.3 ± 2.5
**Top-10% pooling**	99.1 ± 0.8	15.9 ± 4.7	15.0 ± 3.9	99.3 ± 0.8	19.6 ± 6.3	18.9 ± 5.5
**Top-20% pooling**	98.6 ± 0.9	30.7 ± 0.8	29.3 ± 1.4	98.7 ± 1.1	31.3 ± 9.5	30.0 ± 8.4
**Top-30% pooling**	97.8 ± 1.4	37.1 ± 0.5	34.9 ± 1.0	97.9 ± 1.8	41.8 ± 10.5	39.7 ± 8.8
**Maximum pooling**	100.0 ± 0.0	4.6 ± 2.2	4.6 ± 2.2	99.8 ± 0.2	5.7 ± 2.8	5.5 ± 2.7
**10% trimmed mean**	87.1 ± 3.4	79.5 ± 6.2	66.6 ± 9.2	84.5 ± 5.6	84.6 ± 7.9	69.1 ± 2.3

Values are means ± sample standard deviations across the first three repeated sow-level splits. Sensitivity and specificity are expressed as percentages. Youden's index was calculated separately for each split and is reported on a scale from −100 to 100. Minor discrepancies between the displayed mean Youden's index and mean sensitivity (%) + mean specificity (%) − 100 reflect rounding.

Supplementary Table S6

Table S6. Video-level performance of three backbones under four experimental conditions over days 18–25 using the first three repeated sow-level splits.

Backbone	Condition	Accuracy	Sensitivity	Specificity	F1-score	Youden's index	AUC
**Dataset A**							
**Xception**	Original	82.6 ± 1.7	86.3 ± 3.5	74.2 ± 12.1	87.3 ± 0.8	60.5 ± 8.9	0.899 ± 0.024
	Preprocessing	82.3 ± 4.8	87.2 ± 2.3	70.9 ± 13.7	87.2 ± 3.1	58.2 ± 14.3	0.881 ± 0.047
	Mixup	85.4 ± 4.0	92.1 ± 4.8	70.3 ± 5.7	89.7 ± 3.0	62.4 ± 8.1	0.912 ± 0.034
	Combined	84.5 ± 3.2	86.3 ± 2.6	80.2 ± 6.1	88.5 ± 2.3	66.5 ± 7.8	0.902 ± 0.021
**ConvNeXt-Tiny**	Original	83.8 ± 0.4	89.4 ± 5.9	71.4 ± 11.7	88.4 ± 0.8	60.8 ± 5.8	0.901 ± 0.028
	Preprocessing	84.9 ± 4.3	86.2 ± 4.6	82.0 ± 4.0	88.7 ± 3.4	68.1 ± 8.4	0.914 ± 0.036
	Mixup	84.6 ± 1.8	93.4 ± 3.5	64.7 ± 13.2	89.3 ± 0.8	58.1 ± 9.9	0.902 ± 0.020
	Combined	84.9 ± 2.6	91.0 ± 5.1	70.9 ± 17.5	89.3 ± 1.4	61.9 ± 13.0	0.911 ± 0.045
**ViT-Small**	Original	84.5 ± 3.0	91.3 ± 4.4	69.1 ± 15.2	89.1 ± 1.9	60.5 ± 12.1	0.919 ± 0.024
	Preprocessing	81.5 ± 5.5	96.3 ± 4.0	48.3 ± 26.8	87.9 ± 2.8	44.5 ± 22.8	0.908 ± 0.032
	Mixup	82.9 ± 5.1	90.6 ± 2.3	65.6 ± 15.5	88.1 ± 3.3	56.2 ± 15.9	0.890 ± 0.049
	Combined	83.3 ± 3.7	91.2 ± 1.2	65.3 ± 14.7	88.3 ± 2.1	56.5 ± 13.6	0.884 ± 0.043
**Dataset B**							
**Xception**	Original	83.0 ± 2.3	84.1 ± 10.3	81.2 ± 11.2	86.0 ± 3.0	65.3 ± 1.6	0.919 ± 0.017
	Preprocessing	82.2 ± 2.7	82.0 ± 9.9	82.7 ± 11.2	85.1 ± 3.2	64.7 ± 3.9	0.916 ± 0.021
	Mixup	84.6 ± 3.5	83.9 ± 9.9	86.0 ± 9.8	87.1 ± 3.8	69.9 ± 4.7	0.937 ± 0.010
	Combined	84.5 ± 0.6	84.3 ± 5.5	85.1 ± 7.7	87.2 ± 1.1	69.3 ± 2.4	0.928 ± 0.004
**ConvNeXt-Tiny**	Original	84.2 ± 3.8	90.2 ± 6.0	73.8 ± 20.4	87.8 ± 1.8	64.1 ± 14.5	0.928 ± 0.021
	Preprocessing	86.3 ± 2.0	89.7 ± 5.3	80.5 ± 11.3	89.1 ± 1.4	70.2 ± 6.9	0.938 ± 0.018
	Mixup	86.8 ± 3.1	90.7 ± 2.8	80.3 ± 3.7	89.6 ± 2.6	70.9 ± 6.4	0.939 ± 0.018
	Combined	86.0 ± 1.5	89.1 ± 2.5	80.8 ± 7.5	88.8 ± 0.9	69.8 ± 5.2	0.931 ± 0.008
**ViT-Small**	Original	83.7 ± 3.3	89.1 ± 7.4	74.7 ± 15.9	87.2 ± 2.4	63.8 ± 10.5	0.926 ± 0.018
	Preprocessing	82.0 ± 3.9	92.8 ± 3.5	63.8 ± 15.8	86.7 ± 2.2	56.7 ± 12.7	0.909 ± 0.020
	Mixup	83.6 ± 1.9	89.8 ± 2.8	73.4 ± 9.6	87.3 ± 1.0	63.2 ± 6.9	0.912 ± 0.010
	Combined	83.5 ± 2.3	90.4 ± 5.7	71.8 ± 6.1	87.2 ± 2.2	62.2 ± 3.5	0.908 ± 0.016

Values are means ± standard deviations across the first three shared sow-level splits. Xception results are restricted to these splits to match the additional-backbone comparisons. Preprocessing denotes wavelet denoising followed by CLAHE, and Combined denotes preprocessing with Mixup. Youden's index is reported on a scale from −100 to 100. Differences between displayed means are descriptive and do not establish statistical superiority.

Table S6 compares Original, Preprocessing, Mixup and Combined across three backbones using the first three shared sow-level splits. Relative to Original, Combined yielded higher mean accuracy and Youden's index for both Xception and ConvNeXt-Tiny in both datasets. For ConvNeXt-Tiny, mean accuracy increased from 83.8% to 84.9% in Dataset A and from 84.2% to 86.0% in Dataset B, while Youden's index increased from 60.8 to 61.9 and from 64.1 to 69.8, respectively. These results extend the descriptive evidence of benefits over Original to an additional CNN configuration. However, Combined was not consistently the best-performing condition: for ConvNeXt-Tiny, Preprocessing yielded the highest mean Youden's index and AUC in Dataset A, whereas Mixup yielded the highest mean accuracy, Youden's index and AUC in Dataset B.

The benefits observed with the CNN configurations were not uniformly reproduced with ViT-Small. Compared with Original, Combined yielded lower mean accuracy, specificity, Youden's index and AUC in both datasets. For example, mean AUC decreased from 0.919 to 0.884 in Dataset A and from 0.926 to 0.908 in Dataset B. In Dataset B, mean sensitivity increased from 89.1% to 90.4%, while specificity decreased from 74.7% to 71.8%,

illustrating a different error balance rather than an overall improvement. Thus, the additional comparisons provide some support for Combined beyond Xception, but also show that its benefits depend on the evaluated backbone and dataset. These findings concern the specific configurations tested: input sizes differed between backbones, and shared training settings were used rather than architecture-specific optimization. With only three repeated splits and no paired uncertainty estimates reported for these additional-backbone comparisons, the mean differences should be interpreted descriptively and do not establish statistical superiority or explain the mechanisms underlying the different responses.

Supplementary Table S7

Table S7 compares wavelet denoising, CLAHE and their sequential application without Mixup using the first three repeated splits. In Dataset A, both individual components yielded higher mean accuracy and sensitivity than Original, but lower specificity and AUC. Mean Youden's index was slightly lower with wavelet denoising and slightly higher with CLAHE. Thus, the accuracy increases were not accompanied by consistent improvements across the other metrics.

In Dataset B, wavelet denoising yielded higher mean accuracy, Youden's index and AUC than Original. CLAHE yielded the highest mean specificity and Youden's index among the four conditions, increasing specificity from 81.2% to 90.3% while reducing sensitivity from 84.1% to 80.0%. The effects of the individual components therefore differed between datasets.

Under the evaluated settings without Mixup, sequential application of wavelet denoising and CLAHE did not yield higher mean accuracy, Youden's index or AUC than either component alone in either dataset. Its mean values for these three metrics were also slightly lower than those of Original in both datasets, providing no numerical evidence of an advantage for sequential preprocessing in these comparisons. This comparison does not identify which image changes were responsible for the observed performance differences.

Table S7. Video-level comparison of wavelet denoising and CLAHE without Mixup over days 18–25 using the first three repeated splits.

Dataset	Condition	Accuracy	Sensitivity	Specificity	F1-score	Youden's index	AUC
A	Original	82.6 ± 1.7	86.3 ± 3.5	74.2 ± 12.1	87.3 ± 0.8	60.5 ± 8.9	0.899 ± 0.024
	Wavelet only	83.6 ± 3.4	89.5 ± 6.8	70.4 ± 12.5	88.3 ± 2.6	59.9 ± 9.3	0.889 ± 0.038
	CLAHE only	84.1 ± 3.8	89.8 ± 2.7	71.4 ± 9.5	88.7 ± 2.6	61.2 ± 10.5	0.890 ± 0.044
	Preprocessing	82.3 ± 4.8	87.2 ± 2.3	70.9 ± 13.7	87.2 ± 3.1	58.2 ± 14.3	0.881 ± 0.047
B	Original	83.0 ± 2.3	84.1 ± 10.3	81.2 ± 11.2	86.0 ± 3.0	65.3 ± 1.6	0.919 ± 0.017
	Wavelet only	83.8 ± 4.8	84.4 ± 14.5	82.7 ± 11.4	86.3 ± 5.8	67.1 ± 3.1	0.933 ± 0.013
	CLAHE only	83.8 ± 2.0	80.0 ± 2.8	90.3 ± 2.6	86.1 ± 1.9	70.3 ± 3.9	0.927 ± 0.013
	Preprocessing	82.2 ± 2.7	82.0 ± 9.9	82.7 ± 11.2	85.1 ± 3.2	64.7 ± 3.9	0.916 ± 0.021

Values are mean ± standard deviation (SD) across the first three repeated splits. Preprocessing denotes wavelet denoising followed by CLAHE; Mixup was not applied in any condition. Accuracy, sensitivity, specificity and F1-score are expressed as percentages. Youden's index is reported on a scale from −100 to 100.

Supplementary Table S8

Table S8 summarizes the video-level test performance of Xception with letterbox resizing across the first three repeated sow-level splits. The corresponding direct-resizing baseline is reported for Xception under Original in Table S6. Letterbox models were trained using the same data partitions.

During testing, the existing masking step was retained, and only the final direct resizing was replaced with letterbox resizing to 299 × 299 pixels. The same test videos and video-level decision rule were used as for Original. The 18–25-day restriction applies only to pregnant-sow videos; all corresponding held-out nonpregnant videos were included.

Table S8. Video-level performance of Xception with letterbox resizing over days 18–25 using the first three repeated sow-level splits.

Dataset	Accuracy	Sensitivity	Specificity	F1-score	Youden's index	AUC
A	82.5 ± 0.1	90.8 ± 3.6	63.6 ± 8.7	87.8 ± 0.4	54.5 ± 5.1	0.886 ± 0.011
B	81.3 ± 3.8	84.7 ± 10.8	75.6 ± 11.3	84.8 ± 4.3	60.3 ± 5.8	0.902 ± 0.022

Values are means ± sample standard deviations (SDs) across the first three repeated sow-level splits (split-1–split-3). Accuracy, sensitivity, specificity and F1-score are expressed as percentages; Youden's index is reported on a scale from −100 to 100. AUC was calculated using the proportion of frames classified as pregnant as the video-level score.

Supplementary Figure S1

Gradient-weighted class activation mapping (Grad-CAM) [36] was used to qualitatively examine four selected split-1 test videos: a true positive from Dataset A at day 25, a true negative from Dataset B, a false positive from Dataset A and a false negative from Dataset B at day 21. Each video had the same video-level classification outcome under all four conditions. Frame index 50 (zero-based; approximately 5 s) was used for every case. Maps were generated from the conv4.pointwise layer of Xception using the corresponding checkpoints and evaluation preprocessing. The target was the shared video-level prediction: Pregnant for true-positive and false-positive cases, and Nonpregnant for true-negative and false-negative cases.

Figure S1 illustrates differences in spatial Grad-CAM responses across conditions even when the video-level classification outcome was identical, including in the consistently misclassified videos. In the false-positive case, Original classified the selected frame as Nonpregnant despite the video-level Pregnant prediction. Thus, the case labels describe video-level outcomes, whereas the maps describe target-class responses for individual frames. A frame from a pregnant video need not contain visible pregnancy-related structures.

These examples illustrate model responses but do not establish anatomical localization or the causes of classification errors. Because these examples were selected to have identical video-level outcomes across conditions, they cannot explain between-condition differences in classification performance. Independent min–max normalization also prevents comparison of absolute response magnitudes across maps.

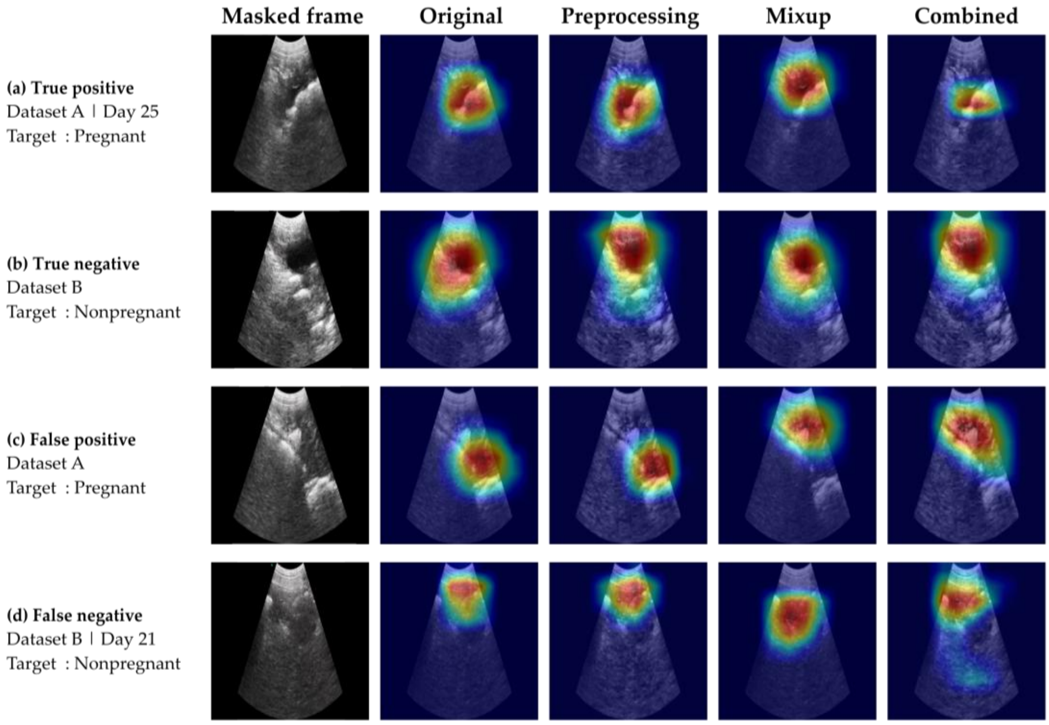

Figure S1. Grad-CAM maps for selected (a) true-positive, (b) true-negative, (c) false-positive and (d) false-negative videos at frame index 50. Columns show the masked frame and the four experimental conditions. Maps are independently min–max normalized; warmer colors indicate higher relative activation within each map.

Reference numbering follows the main manuscript.

References

36. Selvaraju, R.R.; Cogswell, M.; Das, A.; Vedantam, R.; Parikh, D.; Batra, D. Grad-CAM: Visual Explanations from Deep Networks via Gradient-Based Localization. *International Journal of Computer Vision* **2020**, *128*, 336–359. 10.1007/s11263-019-01228-7.
